# Identification of avian flapping motion from non-volant winged dinosaurs based on modal effective mass analysis

**DOI:** 10.1371/journal.pcbi.1006846

**Published:** 2019-05-02

**Authors:** Yaser Saffar Talori, Jing-Shan Zhao, Yun-Fei Liu, Wen-Xiu Lu, Zhi-Heng Li, Jingmai Kathleen O'Connor

**Affiliations:** 1 Department of Mechanical Engineering, Tsinghua University, Beijing, P. R. China; 2 Key Laboratory of Vertebrate Evolution and Human Origins, Institute of Vertebrate Paleontology and Paleoanthropology, Chinese Academy of Sciences, Beijing, P. R. China; Universidade Federal de Santa Catarina, BRAZIL

## Abstract

The origin of avian flight is one of the most controversial debates in Paleontology. This paper investigates the wing performance of *Caudipteryx*, the most basal non-volant dinosaur with pennaceous feathered forelimbs by using modal effective mass theory. From a mechanical standpoint, the forced vibrations excited by hindlimb locomotion stimulate the movement of wings, creating a flapping-like motion in response. This shows that the origin of the avian flight stroke should lie in a completely natural process of active locomotion on the ground. In this regard, flapping in the history of evolution of avian flight should have already occurred when the dinosaurs were equipped with pennaceous remiges and rectrices. The forced vibrations provided the initial training for flapping the feathered wings of theropods similar to *Caudipteryx*.

## Author summary

The origin of avian flight in the perspective of mechanics has been investigated for the first time. We reported the first evidence for flapping hypothesis based on principle of physical modeling. This is significant because using modal effective mass method and reconstructed *Caudipteryx*, the most basal non-volant winged dinosaur, we captured significant and negligible modes and realized that resonance oscillation of *Caudipteryx* wings could occur as the running speed approached to the primary frequencies. Such forced vibrations induced by legs' motions during running trained the *Caudipteryx* and the other feathered dinosaurs to flap their wings.

## Introduction

The origin of avian flight has been debated for over 150 years, ever since the discovery of the first fossil of *Archaeopteryx* in 1861 [1–35]. Being widely considered as the oldest and most basal-known avian taxon, *Archaeopteryx* is characterized by a long boney tail, three clawed digits forming the manus, teeth throughout the upper and lower jaws, a furcula, a non-ossified sternum, and perhaps most importantly, forelimbs with elongate asymmetrical feathers forming large wings. It is widely accepted that birds are nestled within the derived lineage of theropod dinosaurs, the *Maniraptora*. However, it is still subject to heavy debate how flight evolved within the *Dinosauria*, and multiple origins of flight appear increasingly probable [1–12, 24, 31]. Many researchers consider that avian flight evolved through a number of stages from a ground-dwelling quadrupedal reptile [14–18], cursorial bipedal ground-dweller [13–17, 19], and arboreal life [14, 20] including parachuting [14, 21, 22], gliding [14, 16, 20, 22, 23], and eventually achieving active powered flapping flight [14, 16, 20–23]. However, there is increasing support from studies of juvenile birds for a ground up hypothesis in which flight evolved in a terrestrial animal and the flight stroke evolved directly without an intervening gliding phase [36–43]. Among non-avian dinosaurs [20], *Caudipteryx* represents the most basal taxon with almost completely preserved feathered forelimbs that could be considered ‘proto-wings’ making this taxon important to the study [21] of flight origins [15, 22, 26]. Some other non-volant theropods from the Cretaceous period have been reported with feathered forelimbs [27, 28]. *Caudipteryx* is a basal member of the *Pennaraptora* [1], a derived group of maniraptoran dinosaurs, sometimes closely allied with birds and the most primitive group with pennaceous feathers. *Caudipteryx* has short forelimbs with distally located symmetrical pennaceous feathers and long hindlimbs. The feathering of both fore-and hindlimbs indicates that *Caudipteryx* was not a volant theropod [26]. *Caudipteryx* further differs from modern birds which have abbreviated tails and forward centered mass locating near the wings [29]. However, the most primitive winged dinosaur, *Caudipteryx*, is clearly terrestrial, investigating the aerodynamic properties of the proto-wings of *Caudipteryx* has the potential to shed light on the origin of avian flight [30].

We estimated the maximum running speed of *Caudipteryx* to be about 8 m/s. This value was based on the skeletal hindlimb proportions of BPM 00001 and on adopting the assumptions [44, 45] with respect to the limb posture of small theropods and the range of Froude numbers (up to 17) they might have utilized in running (see S1 Speed for detailed calculation about speed) [44, 45]. We also focused our analysis on a literally generic *Caudipteryx* with a body mass of 5 kg, a realistic value given that an empirical equation for estimating theropod body masses on the basis of femoral length [46] produces results ranging from 4.74 kg to 5.18 kg (mean value = 4.96 kg) for a total of five described specimens (see S1 Mass for detailed calculation about mass) [24, 47, 48].

Any part, mechanism or system has its particular natural frequencies and corresponding mode shapes [46–51]. Mathematically we can compute which natural frequency and related mode shape is significant and effective to take them into account [49, 52]. The theory of modal effective mass is based on natural frequency, modal analysis and effective masses associated with different directions [49]. The modal effective mass is a measure to classify the importance of a mode shape when a structure is excited by the enforced acceleration from base. A high effective mass in a certain direction will lead to a high reaction force at the base and will be easily excited. Resonance phenomenon occurs on the *Caudipteryx* when the frequency of the forced vibrations excited by running legs is matched with any natural frequency of *Caudipteryx*. Hence, by detecting effective natural frequencies of the whole body and analysis of corresponding mode shapes, the velocities of the *Caudipteryx* that stimulate the wings to flap can be obtained (50 cm is measured for the step length of *Caudipteryx*).

To this end, a simplified mathematical model, a Finite Element Model, a reconstructed physical model of *Caudipteryx*, and experiment on young ostrich have been utilized. The simplified mathematics model helps us to understand how to face with the kinematics of *Caudipteryx*. Finite Element (FE) model gives a precise and acceptable result to compare with the reconstructed model on the test rig and experiment on running juvenile ostrich proves the mathematical analyses and simulations.

## Methods

### Ethics statement

All experiments using juvenile ostriches, data collection and data analysis procedures in this research were carried out in full accordance with ethical rules for animal welfare and according to the requirements of the Ethics Committee of Tsinghua University.

### Modal effective mass and kinematics of *Caudipteryx* (mathematical model)

Effective mass categorizes the significance of a mode shape while a structure is excited by forced vibrations from the base. A higher effective mass will certainly lead to a higher reaction force from the basis, while mode shapes with lower related modal effective masses are hardly excited by base vibration and will provide lower reaction forces at the basis [49–55] (see S1 Text for detailed explanations about modal effective mass method). The analyses using the theory of modal effective masses represent that at which velocities, *Caudipteryx* could most obviously sense flapping on its wings and shoulder joints. This phenomenon is purely governed by the natural biophysics.

#### Seven-degree-of-freedom system of simplified *Caudipteryx*’s body mass

Any system under free vibration oscillates at its natural frequencies which are properties of the dynamical system established by its mass and stiffness distribution. When the external force excitation is oscillatory, the system is forced to oscillate at the excitation frequency and if this frequency coincides with one of the natural frequencies of the system, a condition of resonance is encountered. As energy is dissipated by friction and damping, vibrating systems are all subject to damp. The small values of damping have very little influence on the natural frequencies of the system, therefore, the computations of the natural modes are generally made on the basis of no damping. Equation (S29) and Fig C in S1 Text with considering damping in a system illustrate that damper decreases the amplitude of natural frequency vibration to fully damp the whole system (damping is limiting the amplitude of vibration at resonance). Hence, in an oscillating system, small value of damping ratio (less than 10%) almost cannot effect on free natural vibration and it only reduces the amplitude of vibration in long term [49] (see S1 Text for detailed explanations about vibration theory).

**Fig 1:**
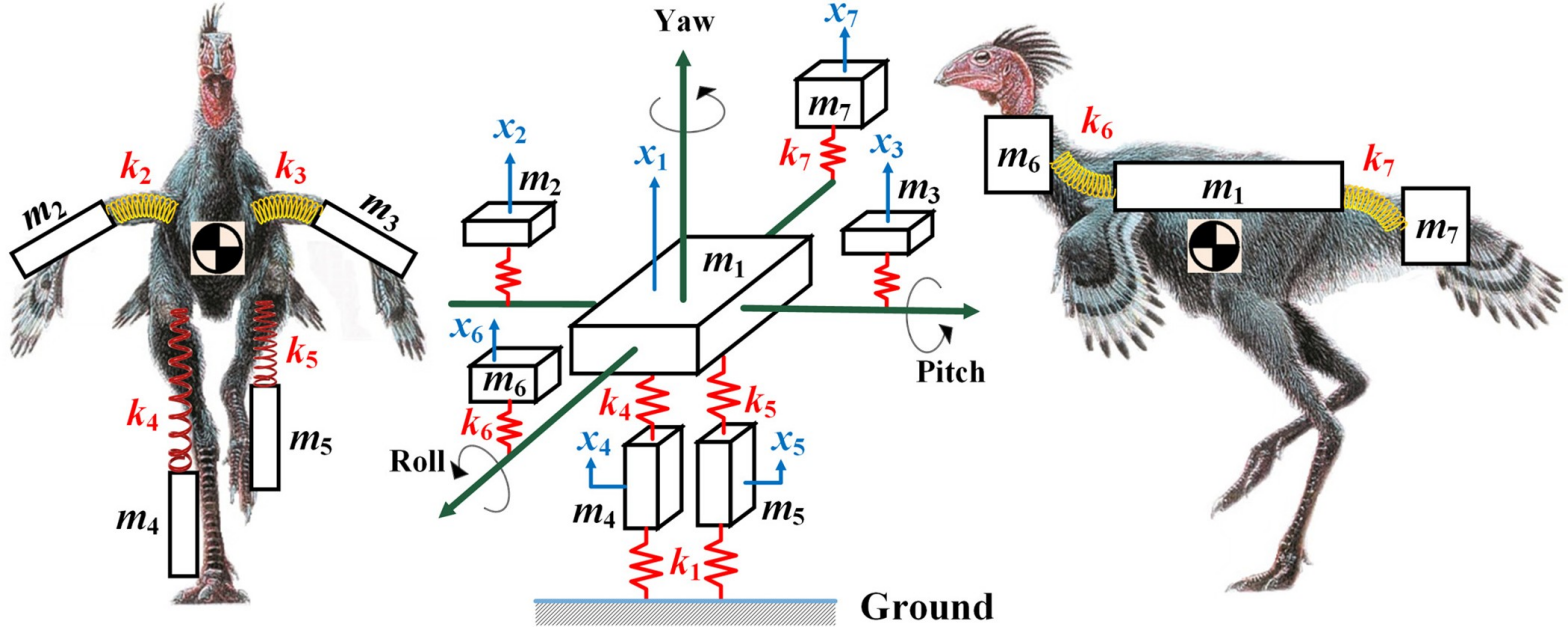
Seven-rigid-body system of *Caudipteryx*. The simplified rigid body system illustrates the mechanism of moving parts, main body, wings, legs, neck and head, and the tail of the *Caudipteryx*. The masses of all parts are represented by lumped mass points and the muscles at the joints are replaced with springs (As damping coefficient does not significantly affect the natural frequency, we simplified the joints which are composed of tendons, muscles, ligaments and soft tissues as purely elastic springs with no damping). Different effective masses of these seven primary modes of the simplified *Caudipteryx* show different possibilities to be excited.

In order to find out *Caudipteryx*’s natural frequencies, related mode shapes and effective masses, a simplified seven-degree-of-freedom rigid body system was first established (Fig 1). Modes with relatively high effective masses are readily excited by running stimulation. However, the modes with low effective masses cannot be readily excited in this manner. This theoretical model divided *Caudipteryx* into seven elements/masses (body, two legs, two wings, tail, and neck and head) and as the excitation from the basis (running legs) is supposed in vertical direction, the response will be expected in the same direction with excitation (it is shown in the precise FE model of *Caudipteryx* that there is no effective mass in lateral motions in any mode). Hence, in this model, the degree of freedom (DOF) for each element/mass was defined solely in vertical direction (namely ***x***_1_,***x***_2_,…,***x***_7_). It is a relatively rough estimation of natural frequencies, mode shapes and effective masses to divide the whole body of *Caudipteryx* into seven degrees of freedom in vertical direction while ignoring the rolling of the body. The system is excited by the displacements of walking feet, *x*_4_ and *x*_5_. After these excitations the whole body masses (Table B in S1 Table) move along their individual directions. The homogeneous equation of the lumped-mass system dynamics is expressed as [49, 52].

$$M_{7\times 7}{\ddot{x}}_{7\times 1}+C_{7\times 7}{\dot{x}}_{7\times 1}+K_{7\times 7}x_{7\times 1}=F$$ (1)

where **M**_7×7_ is the lumped mass matrix, **C**_7×7_ is damping matrix, **K**_7×7_ is the stiffness matrix, ${\ddot{x}}_{7\times 1}$ is the acceleration vector, ${\dot{x}}_{7\times 1}$ is the velocity vector, ***x***_7×1_ is the displacement vector and ***F*** is the base excitation function. Normal modes (natural frequencies) are free undamped vibrations that depend only on the mass and stiffness of the system and how they are distributed [49]. Therefore, damping and excitation force are not required to be taken into account in Eq (1). Hence the equation is rewritten as

$$M_{7\times 7}{\ddot{x}}_{7\times 1}+K_{7\times 7}x_{7\times 1}=0$$ (2)

Solutions for the homogeneous Eq (2) would be earned in terms of eigenvalues (natural frequencies) and eigenvectors (mode shapes). With the mass distribution of *Caudipteryx* (Fig 1 and Table B in S1 Table), we consider each degree of freedom as a motion in the ***x***_7×1_-direction to simplify the effective modal mass calculation.

The effective mass characterizes the mode and it is independent from the eigenvector normalization. Modal participation factors ***L***_*i*_ is determined by

$$L_{i}=\phi_{i}^{T}M\phi_{r}$$ (3)

where ***ϕ***_***i***_ is the *i*th mode and ***ϕ***_***r***_ is the rigid body.

The generalized effective mass is

$${\tilde{M}}_{i}=\frac{L_{i}^{T}L_{i}}{m_{i}}$$ (4)

where ${\tilde{M}}_{i}$ is the *i*th effective mass and ***m***_*i*_ is the generalized mass of mode *i*.

To establish the kinematic equation, the boundary conditions of the system are set as that

$$\begin{array}{l} m_{1}{\ddot{x}}_{1}=k_{4}\left(x_{4}-x_{1}\right)+k_{5}\left(x_{5}-x_{1}\right)-k_{3}\left(x_{1}-x_{3}\right)-k_{2}\left(x_{1}-x_{2}\right)-k_{6}\left(x_{1}-x_{6}\right)-k_{7}\left(x_{1}-x_{7}\right)\\ m_{2}{\ddot{x}}_{2}=k_{2}\left(x_{1}-x_{2}\right)\\ m_{3}{\ddot{x}}_{3}=k_{3}\left(x_{1}-x_{3}\right)\\ m_{4}{\ddot{x}}_{4}=k_{4}\left(x_{1}-x_{4}\right)-k_{1}x_{4}\\ m_{5}{\ddot{x}}_{5}=k_{5}\left(x_{1}-x_{5}\right)-k_{1}x_{5}\\ m_{6}{\ddot{x}}_{6}=k_{6}\left(x_{1}-x_{6}\right)\\ m_{7}{\ddot{x}}_{7}=k_{7}\left(x_{1}-x_{7}\right) \end{array}$$ (5)

Then the kinematic equations of the system are

$$\begin{array}{l} m_{1}{\ddot{x}}_{1}+\left(k_{2}+k_{3}+k_{4}+k_{5}+k_{6}+k_{7}\right)x_{1}-k_{2}x_{2}-k_{3}x_{3}-k_{4}x_{4}-k_{5}x_{5}-k_{6}x_{6}-k_{7}x_{7}=0\\ m_{2}{\ddot{x}}_{2}-k_{2}x_{1}+k_{2}x_{2}=0\\ m_{3}{\ddot{x}}_{3}-k_{3}x_{1}+k_{3}x_{3}=0\\ m_{4}{\ddot{x}}_{4}-k_{4}x_{1}+\left(k_{1}+k_{4}\right)x_{4}=0\\ m_{5}{\ddot{x}}_{5}-k_{5}x_{1}+\left(k_{1}+k_{5}\right)x_{5}=0\\ m_{6}{\ddot{x}}_{6}-k_{6}x_{1}+k_{6}x_{6}=0\\ m_{7}{\ddot{x}}_{7}-k_{7}x_{1}+k_{7}x_{7}=0 \end{array}$$ (6)

In accordance to Table B in S1 Table and Eqs (2) and (6), the eigenvalues in rad/sec can be found by solving det(***K***−*ω*^2^***M***) = 0. (see S1 Text for detailed explanations about mass and stiffness matrices using *Interval Analysis*.)

$$x={\left[\begin{array}{ccccccc} x_{1} & x_{2} & x_{3} & x_{4} & x_{5} & x_{6} & x_{7} \end{array}\right]}^{T}$$ (7)

$$\ddot{x}={\left[\begin{array}{ccccccc} {\ddot{x}}_{1} & {\ddot{x}}_{2} & {\ddot{x}}_{3} & {\ddot{x}}_{4} & {\ddot{x}}_{5} & {\ddot{x}}_{6} & {\ddot{x}}_{7} \end{array}\right]}^{T}$$ (8)

$$M=\left[\begin{array}{ccccccc} 2.4 & 0 & 0 & 0 & 0 & 0 & 0\\ 0 & 0.5 & 0 & 0 & 0 & 0 & 0\\ 0 & 0 & 0.5 & 0 & 0 & 0 & 0\\ 0 & 0 & 0 & 0.2 & 0 & 0 & 0\\ 0 & 0 & 0 & 0 & 0.2 & 0 & 0\\ 0 & 0 & 0 & 0 & 0 & 0.5 & 0\\ 0 & 0 & 0 & 0 & 0 & 0 & 0.7 \end{array}\right]$$ (9)

$$K=\left[\begin{array}{ccccccc} 3300 & -200 & -200 & -650 & -650 & -800 & -800\\ -200 & 200 & 0 & 0 & 0 & 0 & 0\\ -200 & 0 & 200 & 0 & 0 & 0 & 0\\ -650 & 0 & 0 & 1650 & 0 & 0 & 0\\ -650 & 0 & 0 & 0 & 1650 & 0 & 0\\ -800 & 0 & 0 & 0 & 0 & 800 & 0\\ -800 & 0 & 0 & 0 & 0 & 0 & 800 \end{array}\right]$$ (10)

The eigenvalues in rad/sec are expressed in vector form as below

$$\omega ={\left[\begin{array}{ccccccc} 12.1 & 20 & 23.05 & 36.4 & 47.6 & 90.8 & 92.2 \end{array}\right]}^{T}$$ (11)

Then frequencies in Hz are expressed as

$$f={\left[\begin{array}{ccccccc} 1.924 & 3.18 & 3.67 & 5.8 & 7.57 & 14.45 & 14.7 \end{array}\right]}^{T}$$ (12)

where $f_{i}=\frac{\omega_{i}}{2\pi },\;i=1,2,\cdots ,7$.

The eigenvector matrix is

$$\Phi =\left[\begin{array}{ccccccc} 0.393 & 0 & 0.256 & 0.146 & 0.4 & 0 & -0.12\\ 0.62 & -1 & -0.77 & -0.06 & -0.09 & 0 & 0\\ 0.62 & 1 & -0.77 & -0.06 & -0.09 & 0 & 0\\ 0.158 & 0 & 0.108 & 0.07 & 0.22 & 1.58 & 1.55\\ 0.158 & 0 & 0.108 & 0.0 & 0.22 & -1.58 & 1.55\\ 0.432 & 0 & 0.38 & 0.85 & -0.97 & 0 & 0.03\\ 0.45 & 0 & 0.48 & -0.91 & -0.41 & 0 & 0.02 \end{array}\right]$$ (13)

The eigenvectors could be normalized so that the generalized mass is an identity matrix.

$$\overset{\wedge }{m}=\Phi^{T}M\Phi =\left[\begin{array}{ccccccc} 1 & 0 & 0 & 0 & 0 & 0 & 0\\ 0 & 1 & 0 & 0 & 0 & 0 & 0\\ 0 & 0 & 1 & 0 & 0 & 0 & 0\\ 0 & 0 & 0 & 1 & 0 & 0 & 0\\ 0 & 0 & 0 & 0 & 1 & 0 & 0\\ 0 & 0 & 0 & 0 & 0 & 1 & 0\\ 0 & 0 & 0 & 0 & 0 & 0 & 1 \end{array}\right]$$ (14)

The coefficient vector $\bar{L}$ is

$$\bar{L}=\Phi^{T}M\bar{r}={\left[\begin{array}{ccccccc} 2.158 & 0 & 0.40 & 0.10 & 0.19 & 0 & 0.36 \end{array}\right]}^{T}kg$$ (15)

where $\bar{r}={\left[\begin{array}{ccccccc} 1 & 1 & 1 & 1 & 1 & 1 & 1 \end{array}\right]}^{T}$ can be introduced as an influence vector that shows the displacements of the lumped masses.

The modal participation factor Γ_*i*_ for mode *i* is written as

$$\Gamma_{i}=\frac{{\bar{L}}_{i}}{{\overset{\wedge }{m}}_{ii}}$$ (16)

Hence, the modal participation vector is

$$\Gamma ={\left[\begin{array}{ccccccc} 2.158 & 0 & 0.40 & 0.10 & 0.19 & 0 & 0.36 \end{array}\right]}^{T}$$ (17)

Both coefficient vector $\bar{L}$ and modal participation vector Γ could be identical because of the generalized mass matrix. The modal effective mass ***m***_*eff*,*i*_ for mode *i* is

$$m_{eff,i}=\frac{{\bar{L}}_{i}^{2}}{{\overset{\wedge }{m}}_{ii}}$$ (18)

The effective mass regarding to the related natural frequency presents the possibility of the exiting vibrations in running (Table C in S1 Table). Therefore, the summation of the effective masses equals the total mass of the seven-degree-of-freedom system.

$$m_{eff,1}+m_{eff,2}+m_{eff,3}+m_{eff,4}+m_{eff,5}+m_{eff,6}+m_{eff,7}=5\;kg$$ (19)

### Finite element model of *Caudipteryx* (FE model)

**Fig 2:**
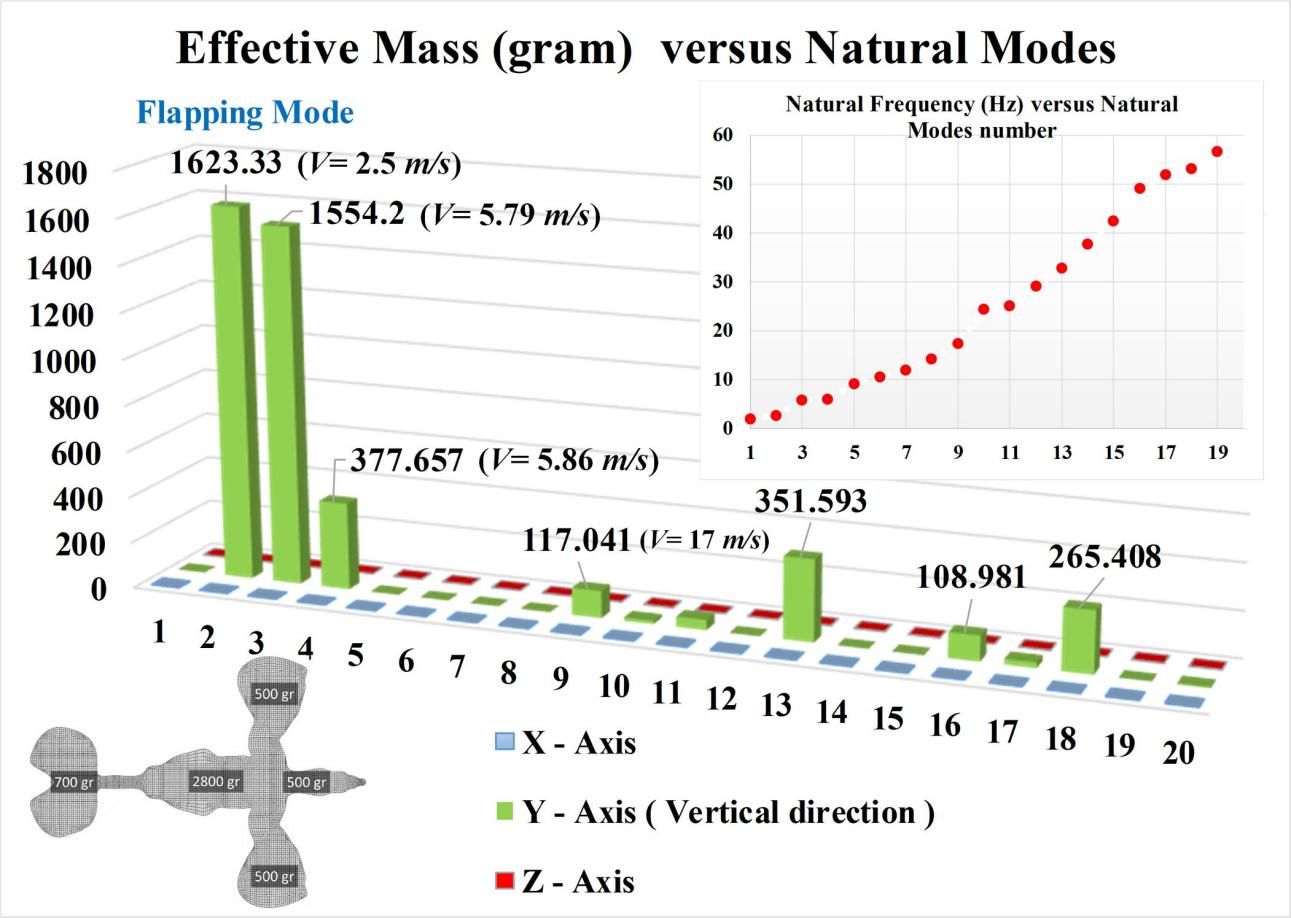
Modal effective mass of *Caudipteryx*. FE model of *Caudipteryx* by using modal effective mass illustrates that the most obvious flapping modes are occurred at the speeds of 2.50 m/s and 5.79 m/s. Y-axis is in the vertical direction and X and Z axes are in lateral directions.

Finite Element Model of *Caudipteryx* provided a precise analysis as the number of elements were sufficient enough and non-structural masses to cover the whole body mass to reach to 5 Kg were also taken into account. Also, except those elements which have boundary conditions, all elements have full DOF in any direction (Fig 2 and S1 Fig).

### Experiments with reconstructed wings on the test rig with *Caudipteryx* robot and Ostrich

We reconstructed the real-sized robot of *Caudipteryx* on the test rig in accordance with the existing fossils (BPM0001) (S2 Fig). The robot is composed of body, tail, neck, wings and legs, and the skeleton is fabricated from ABS plastic. There is no definite evidence for tertiary feathers of *Caudipteryx*. We therefore only reconstructed the primary and secondary remiges from the feathers of extant birds. Using metal pins, we attached them to the antebrachial to generate artificial articulated wings.

In the reconstructed wings, we imbedded force sensors (S3A Fig) to collect the data of lift and thrust/drag (S3C, S3D, S3E and S3F Fig).

**Fig 3:**
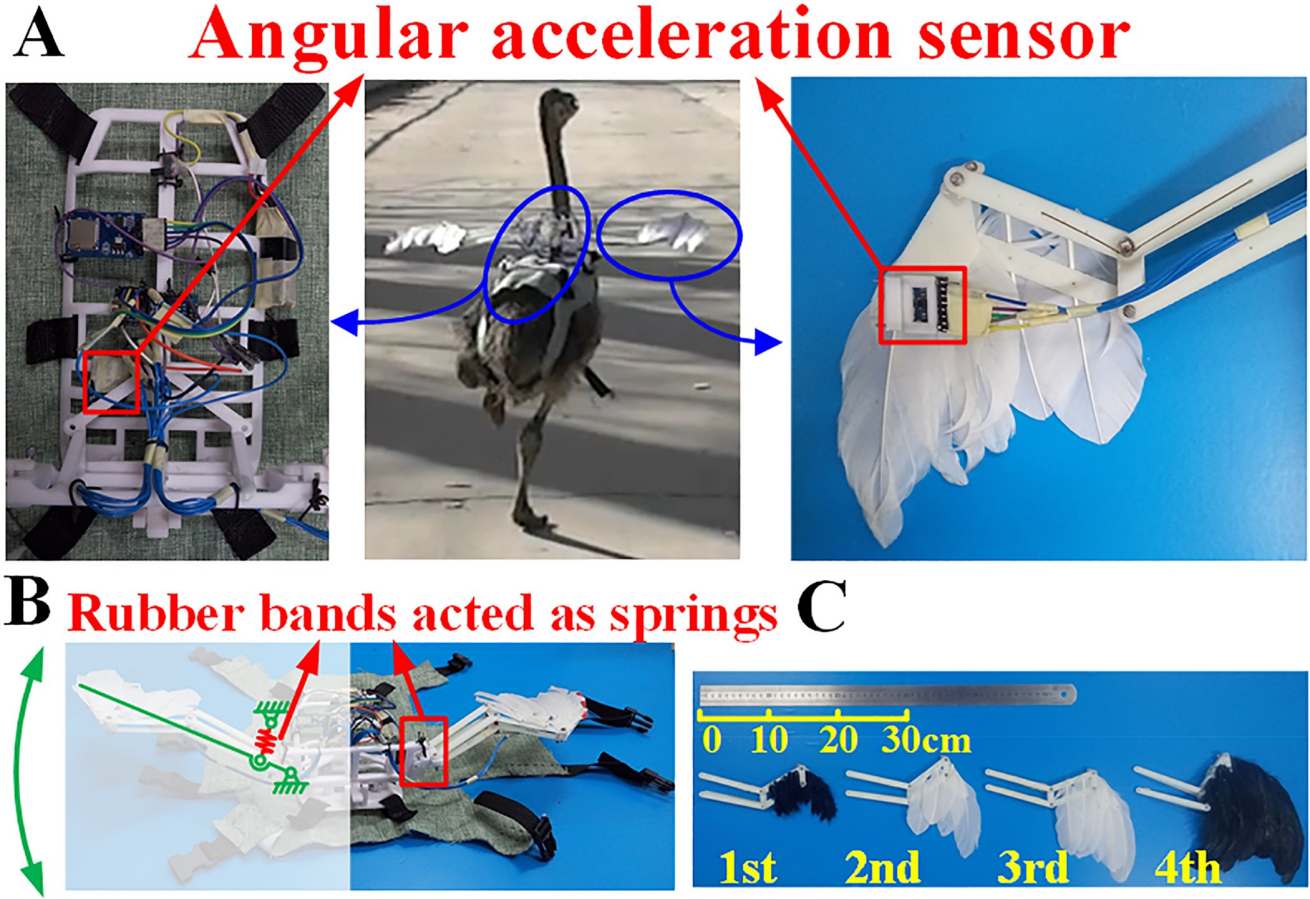
Biophysical vibration of the wings. (A) Wearable devices to detect the performance of wings. The back bracket was manufactured through 3D printer with ABS plastics. The angular accelerometer sensor, force sensor and SD card were all mounted on the bracket (S3B Fig). The accelerometer sensor on the back and the wings were used to measure the rolling angle of body and wings respectively during locomotion on the ground. A force sensor is embedded between the arm and the body to measure the lift generated by the flapping wings (S3A Fig). (B) Simplified wing mechanism. Every wing has a flexible structure that is jointed with the body via elastic rubber belts, which are used to simulate the function of muscles. (C) Reconstruction of wings of different sizes. The first wing represents the forearm with filament feathers. From the second one to the fourth one, the length of feathers increases gradually. The second one represents the short feather, the third one represent middle feather while the fourth one with the longest feathers represents the largest wing (the realistic wing is the third one in accordance to the fossil).

In order to verify experiment by *Caudipteryx* robot and the forced vibrations phenomenon induced by legs, we also implemented the experiment on a half-adulted ostrich whose mass is 6.7 kilograms (Fig 3A) as a similar living bird to *Caudipteryx*. This process was performed through observations on a running juvenile ostrich (S2 Video) and experiments on running ostriches (S3 Video). A device was fixed on the ostrich’s back (Fig 3B) to measure the velocity, acceleration (S3 Fig), rolling angles of body, and wings (S4 Fig). To investigate the responses of the body and the wings in running and the advantage of aerodynamic effects of flapping wings of feathered dinosaurs, we fabricated four different sizes of feathered forearms with the simplest plate wings (Fig 3C) and executed experiments on the ostrich. Therefore, lift and thrust/drag forces produced by artificial wings during running were also measured by the force sensors (S3A and S3B Fig). The connections of the shoulder joints were particularly designed in order to avoid the effect of frictions and inertial forces during locomotion.

## Results

Mathematical model shows the first mode of the forced vibrations to flap the wings when *Caudipteryx* ran on the ground at the speed of about 2 m/s, the mode shape of which is expressed with a vector of ***n***_1_ = (0.393 0.62 0.62 0.158 0.158 0.432 0.45)^T^. The FE model analysis results of the modal effective mass of the *Caudipteryx* (Table E in S1 Table) indicate that the effective natural modes occur only in vertical direction (Y-axis) and they are almost zero in lateral motions (X and Z axes). It expresses that the first natural frequency of about 1.99 Hz is not effective, but the second one of about 2.58 Hz and the third mode of about 5.79 Hz considering the maximum speed of *Caudipteryx* (the forecasted velocity is about 8 m/s for *Caudipteryx*) are effective and important. In other words, the oscillation about the torso axis is the first mode (S1 Fig). Therefore, the *Caudipteryx* should roll its whole body about the torso direction when they ran at a low speed (around 2 m/s) near the first primary frequency. The second primary mode (the most effective mode) occurred as the running speed approached to 2.5 m/s. It means flapping modes were easily excited at low frequency while *Caudipteryx* ran on the ground at the velocity from around 2.5 m/s to a little faster than 5.8 m/s (S1 Fig).

We fabricated four simplest plate wings with different sizes and did experiments on the ostrich to compare the lift forces obtained from the flapping wings passively applied by forced vibrations during running. At the same running speed, the wings with filament feathers (1st wing) provided the smallest lift, the largest value of which is less than 0.13 N, while the ones with longer feathers could provide larger lift (2nd and 3rd wings), and the longest feather (4th wing) could provide the largest lift which exceeds 0.42 N (S3 Fig).

## Discussion

In the simplified rigid body system of seven degrees of freedom of *Caudipteryx*, the whole system can be excited by the displacements of feet, ***x***_4_ and ***x***_5_ during running. After this excitation, the whole body masses move along their individual vertical directions in this model (Fig 1). It illustrates the kinematics of *Caudipteryx* mathematically. In order to obtain the precise results using computer simulation, Finite Element Method reveals the phenomenon that the maximum effective mass occurs in the second mode which is a flapping mode. Only in the most effective mode, could the wings of *Caudipteryx* be excited to flap evidently and then sense lift. Therefore, the results of the FEM model (second model) through Finite Element Method have been considered because of having the highest accuracy. On the other hand, in the FE model simulated by FEM, computer calculations represent that the first natural frequency which had been roughly calculated in the first mathematical model (first model) is almost equal to that of the FEM model; and the other natural frequencies (from the second to the seventh) in comparison with the FEM model (second model) have some deviations but still acceptable. Also in the first model the modal effective masses of each natural mode might not be equal to the accurate FEM model, but the summation of which in simplified seven-degree-of-freedom model must be 5 kilograms. The reason is the limitation on the number of elements/masses (solely seven masses) and having only one DOF in the vertical direction. The effective mass analysis discovers that the first mode has never been effective (Table E in S1 Table). As the speed approached to the second primary frequency, the *Caudipteryx* output the second oscillation mode. It is the flapping of the wings up and down with the same amplitudes and same directions. The simulation has been extended by either increasing or decreasing the mass of each part of the *Caudipteryx* (Table D in S1 Table) and assumed eight excessive masses except the actual one (S5 Fig) (by measuring) from 2 kg to 10 kg in a similar geometrical model. Hence, the frequencies and corresponding effective masses in Y-axis have been studied (Table F in S1 Table). The analyses reveal that the performance of effective modes of any model (models A, B, …, I) are identical but at different frequencies. It means that in all mass distribution models, effective mode mainly depends on the creature’s velocity. When the forced vibration frequency is near the second natural frequency, the flapping mode will be occurred. The natural frequency decreases from 4.0 Hz in mass model A to 1.8 Hz in mass model I (S5 Fig) in the second mode. Therefore, as the weight of the creature increases, the velocity necessary to reach flapping mode might be decreased.

**Fig 4:**
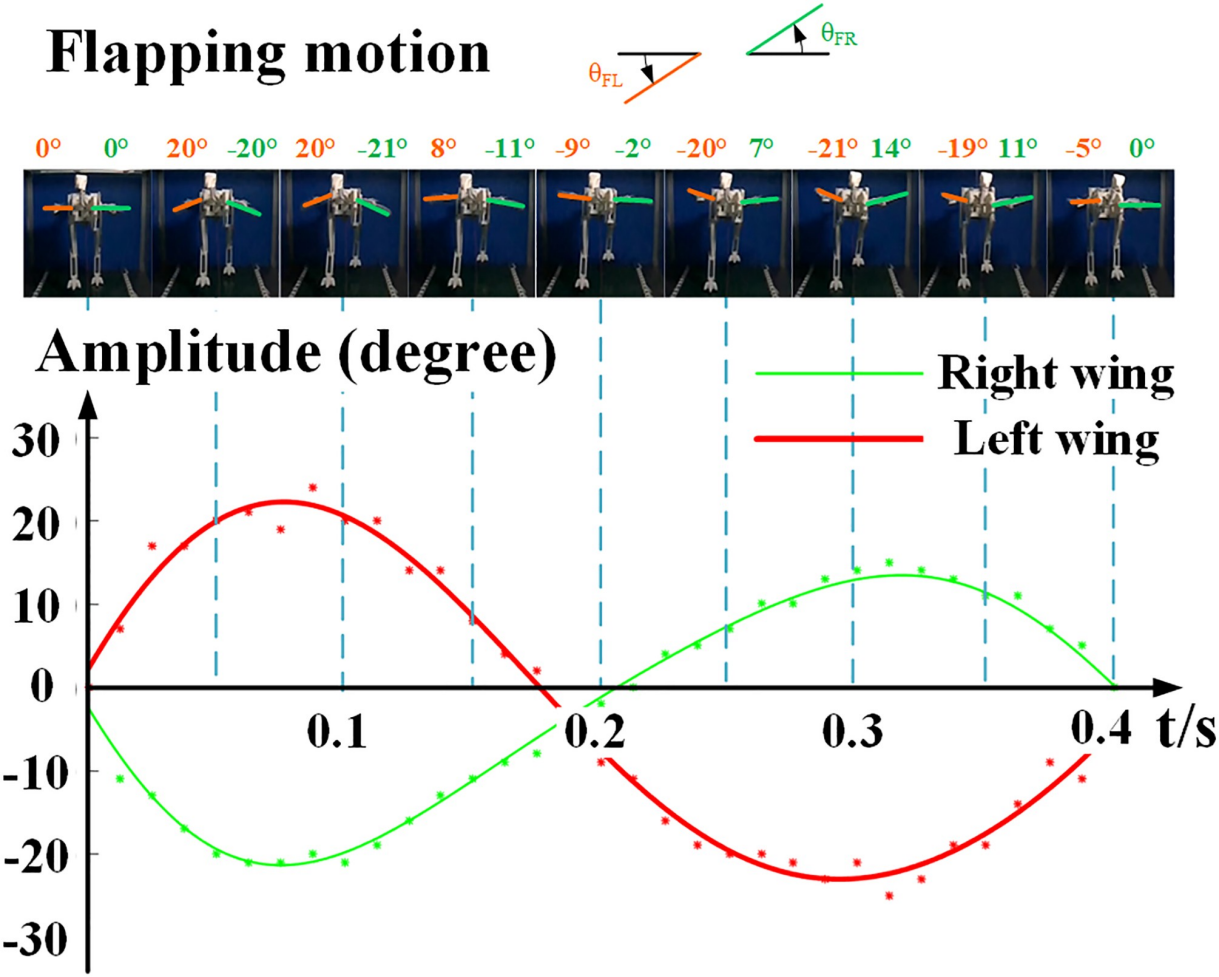
Forced vibrations of wings of *Caudipteryx* robot deduced by test rig (S1 Video) which approaches the flapping flight of modern birds [56]. Through curve iteration, we obtained the flapping function *ϴ*_right_ = 932.7sin(19.01t−3.35)+28.18sin(15.25t−5.103)+898.2 sin(19.16t+6.034) and *ϴ*_left_ = 135.6 sin(6.453t+1.808)+1558 sin(0.4013t+6.198)+6.517 sin(18.87t + 0.4756). We here defined the anticlockwise motion of both wings as the positive direction. Therefore, the down stroke for the left wing is a positive motion while the down stroke for the right wing is a negative one.

With the observation of the experiments, we realized that when the speed of the reconstructed *Caudipteryx* robot on the test rig (S2 Fig) reached 2.31 m/s (near the value of what has been simulated by FEM model), the robot’s wings started to output most obvious flapping motions which is the resonance of forced vibrations in physics (Fig 4). Using theory of modal effective mass and reconstruction of *Caudipteryx zoui* (BPM0001) (S6 Fig and Table A in S1 Table), we infer that flapping flight could be developed earlier than gliding in the evolution of avian flight. When the running speed was near the second primary speed of about 2.5 m/s, both wings of the *Caudipteryx* generated oscillations similar to flapping wings.

Step length in running animals varies with speed and gait and animals do not just have one step length. Any given velocity in this research such as 2, 2.5 and 5.79 m/s dedicated to first, second and third modes was obtained by measuring and assuming some parameters from the fossil such as step length, stiffness and mass (see S1 Text for detailed explanations about *Caudipteryx* velocity and step length). To eliminate these uncertain values, we used interval analysis which is a powerful mathematical tool in engineering (see S1 Text for detailed explanations about Interval Analysis method). Modal effective mass and Interval Analysis represent that flapping motion occurred at lower velocity. It means, if step length was between 30 cm to 70 cm and if mass was between 3 to 7 kg, *Caudipteryx* had flapping motion and it occurred at lower velocities (there must be a value that will render the second mode although we do not know the exact number which is in a certain Interval). Hence, the velocities of 2.5 m/s and 5.86 m/s are only two cases among all possibilities. Therefore, the conclusion that the second and the third modes must occur at a certain value is an objective conclusion. Further, the physical phenomenon of flapping motion (induced by forced harmonious vibrations) always be generated in running, but we cannot obtain the precise value of running speed since it might be expressed with an interval of velocity. Hence, the role of body oscillation during a run should be taken into account in order to understand the origin and evolution of avian flapping wings.

Experiment results on ostrich indicated that the vibrations of the feathered wings were easily induced when ostrich ran on the ground. Under the assumption of the same length of forearms for the feathered dinosaurs, the wing with the shortest feathers generated the flapping motions with the largest amplitude while the ones with longer feathers produced the flapping motions with smaller amplitudes (S4 Fig). This is interpreted by the air resistance. The larger the wing area, the larger the resistance, and the smaller the amplitude for the passive vibrations. This experiment suggests that the flapping motion might be developed by the forced vibrations during terrestrial locomotion when the winged dinosaur appeared on the earth. However, the lift obtained from the running-foot forced vibrations shows that the longer and larger the wing was, the larger the lift would be (S3 Fig). Therefore, forced vibrations may represent the earliest stages in the evolution of forelimb flapping in winged theropods. This suggests that flapping behavior evolved in non-volant theropods long time ago before they could actively fly.

Experiments on the *Caudipteryx* robot based on the fossil (*Caudipteryx* sp. IVPP V12430) and the experiments on artificial wings placed on the back of a juvenile ostrich indicated that the forced vibrations of plumage forearms during walking and running taught the winged theropods to flap their wings. These analyses suggest that the impetus of the evolution of powered flight in the theropod lineage that lead to Aves may have been an entirely natural phenomenon produced by bipedal motion in the presence of feathered forelimbs.

## Supporting information

S1 Fig Computer simulation for the first twenty natural frequencies. Computer simulations were executed at the finite element software of ABAQUS (S4 Video). The basic elements in the simulation were shell, linear quadrilateral with the type of S4R. The first primary mode is the rigid swaying of the wings; the second one is the flapping mode.
(TIF)

S2 Fig Reconstructed *Caudipteryx* robot from fossil BPM 0001. The measurements from *Caudipteryx zoui* BPM 0001 (*Caudipteryx* sp. IVPP V12430) have been used to appraise the whole body of *Caudipteryx* and to characterize the appropriate relationship for the robot and mathematical models of this dinosaur. Every part of the robot was fabricated with 3-D printer, guided by the information from the fossil.
(TIF)

S3 Fig Experiment on the Ostrich to get the lift produced by the artificial wings with the simplest plate form. (A) Force sensor. They are embedded into the wearable device to measure lift dynamically. In this experiment, each wing has one force sensor to measure the dynamic lift. (B) Embedded accelerometer and SD card on a bracket. The accelerometer records the running speed of the ostrich, and collect all experiment data to a micro SD card. (C) Lift from the filament wing. The largest lift of one wing is 0.13 N when the speed is approaching 4 m/s. (D) Lift from the short wing. The largest lift of one wing is about 0.22 N when the speed is approaching 4 m/s. (E) Lift from the middle wing. The largest lift of one wing is about 0.3 N when the speed is around 4 m/s. (F) Lift from the longest wing. The largest lift of one wing is over 0.42 N when the speed exceeds 4m/s. The results show that, at the same speed, longer feather will generate larger lift.
(TIF)

S4 Fig Experiment on the Ostrich to obtain responses of the body and the wings in running. (A) Definition of the flapping angles of the wings. The clockwise rotation is the positive direction for the left wing while the anticlockwise rotation is the positive direction for the right wing. (B) Response of the filament feathers. The largest flapping angle of the wings is around 25°. (C) Response of the short feathers. The largest flapping angle of the wings is about 20°. (D) Response of the middle-sized feathers. The largest flapping angle of the wings is less than 15°. (E) Response of the longest feathers. The largest flapping angle of the wings is less than 10°. This results show that two wings will move up and down simultaneously, which is the flapping motion when the ostrich runs. Longer feather will have smaller flapping angle because of the air resistance during this passive experiment.
(TIF)

S5 Fig Modal effective mass of *Caudipteryx* by means of eight excessive assumed mass distribution. (A) changes of natural frequencies with respect to the modes. (B) effective masses in Y-axis versus modes and velocities to reach to the flapping or second modes. The natural frequency decreases from 4 Hz in mass model A to 1.8 Hz in mass model I in the second mode, hence, as the weight of the creature increases, the velocity in order to reach to the flapping mode might be decreased.
(TIF)

S6 Fig Fossils of *Caudipteryx*. (A) *Caudipteryx dongi* IVPP V12344 and (B) *Caudipteryx* sp. IVPP V12430.
(TIF)

S1 Video Experiments on the test rig. Experiments were in the stationary situation when the robot flapped actively from 2.5 Hertzs to 6.3 Hertzs. Both produced positive lift and thrust forces in this case.
(MP4)

S2 Video Observation on the juvenile ostrich. The forced vibrations of the wings of the young ostriches are easily found when they run on the ground.
(MP4)

S3 Video Experiments on the ostrich. Experiments were accomplished on the ostrich when it ran on the ground with different reconstructed wings of *Caudipteryx*. Collected data show that the flapping of the wings is a natural process of the forced vibrations under the actuation of running feet.
(MP4)

S4 Video Computer simulation on vibration modes of *Caudipteryx*. Computer simulations were also accomplished on reconstructed *Caudipteryx* with wings. Flapping motion is one of the primary modes of the *Caudipteryx* which is easily excited under the actuation of running feet.
(MP4)

S1 Text Supplementary materials. (PDF)

S1 Table Tables. (PDF)

S1 Speed Speed calculation. (XLSX)

S1 Mass Mass calculation. (XLSX)
