# Supplementary figures and images for: Identification of avian flapping motion from non-volant winged dinosaurs based on modal effective mass analysis
Source: PLoS Comput Biol. 2019 May 2;15(5):e1006846. doi: 10.1371/journal.pcbi.1006846 (PMC6497222; doi:10.1371/journal.pcbi.1006846)

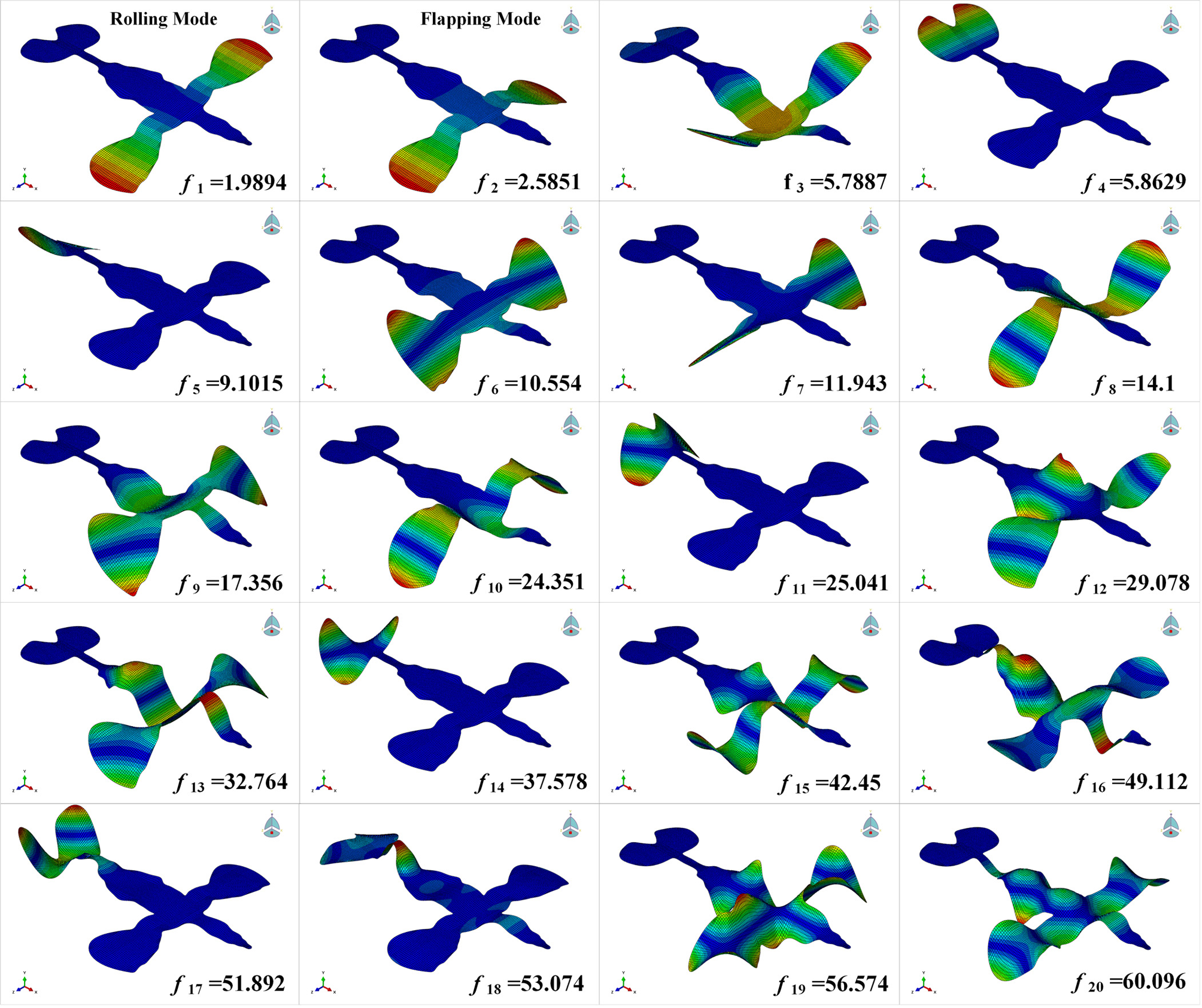

Supplement: S1 Fig — Computer simulations were executed at the finite element software of ABAQUS (S4 Video). The basic elements in the simulation were shell, linear quadrilateral with the type of S4R. The first primary mode is the rigid swaying of the wings; the second one is the flapping mode. (TIF) [file pcbi.1006846.s001.tif]

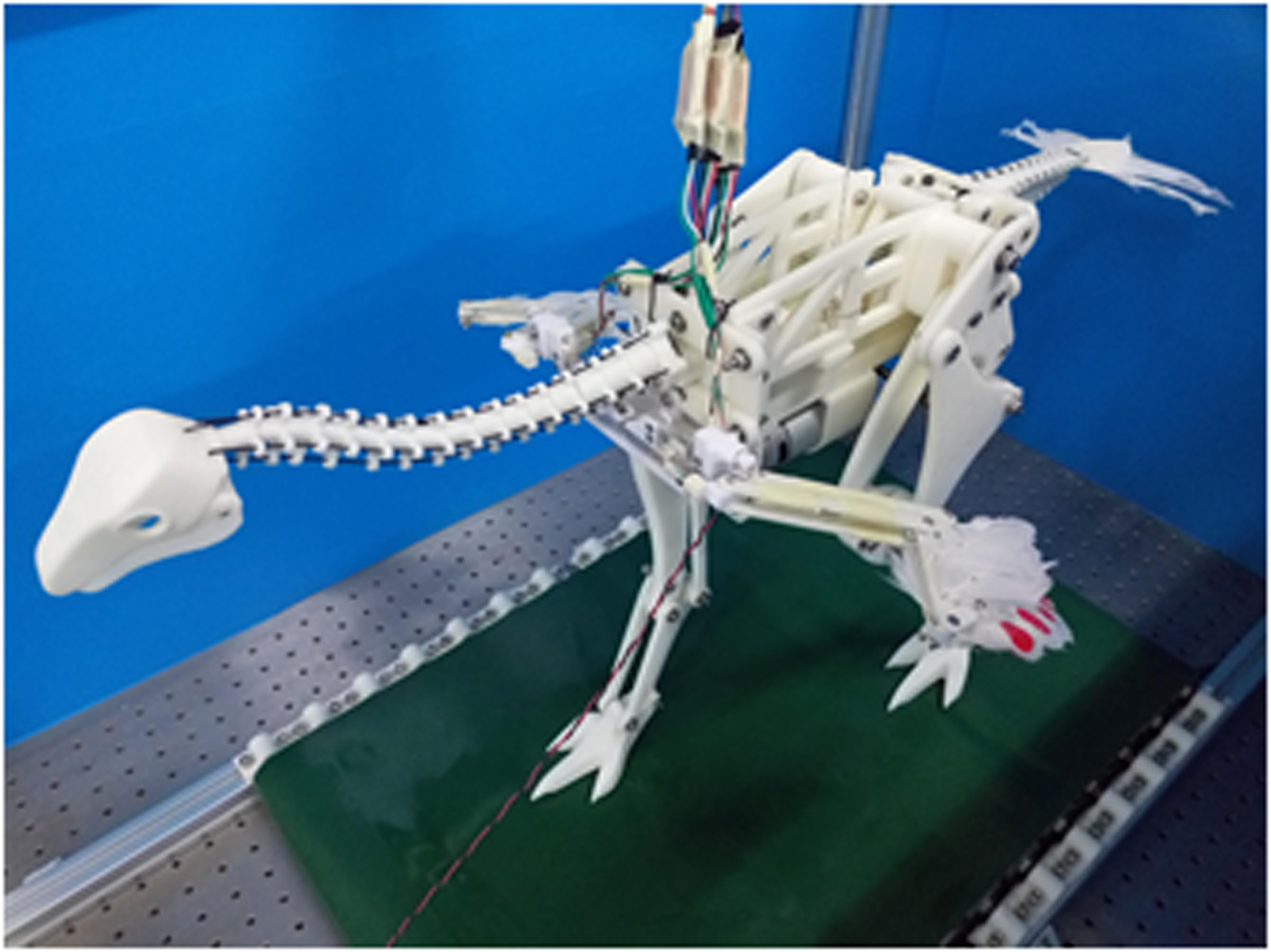

Supplement: S2 Fig — The measurements from Caudipteryx zoui BPM 0001 (Caudipteryx sp. IVPP V12430) have been used to appraise the whole body of Caudipteryx and to characterize the appropriate relationship for the robot and mathematical models of this dinosaur. Every part of the robot was fabricated with 3-D printer, guided by the information from the fossil. (TIF) [file pcbi.1006846.s002.tif]

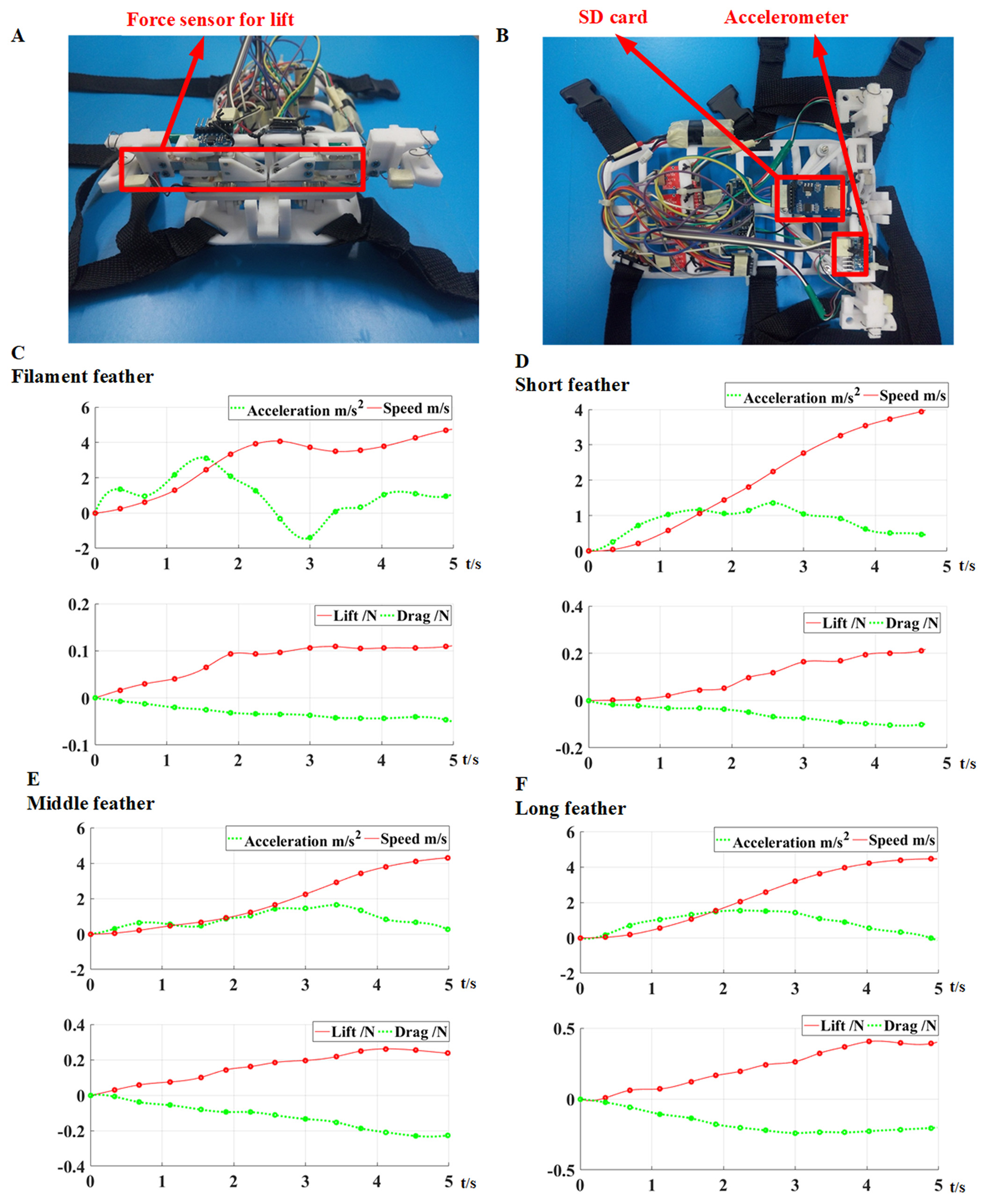

Supplement: S3 Fig — (A) Force sensor. They are embedded into the wearable device to measure lift dynamically. In this experiment, each wing has one force sensor to measure the dynamic lift. (B) Embedded accelerometer and SD card on a bracket. The accelerometer records the running speed of the ostrich, and collect all experiment data to a micro SD card. (C) Lift from the filament wing. The largest lift of one wing is 0.13 N when the speed is approaching 4 m/s. (D) Lift from the short wing. The largest lift of one wing is about 0.22 N when the speed is approaching 4 m/s. (E) Lift from the middle wing. The largest lift of one wing is about 0.3 N when the speed is around 4 m/s. (F) Lift from the longest wing. The largest lift of one wing is over 0.42 N when the speed exceeds 4m/s. The results show that, at the same speed, longer feather will generate larger lift. (TIF) [file pcbi.1006846.s003.tif]

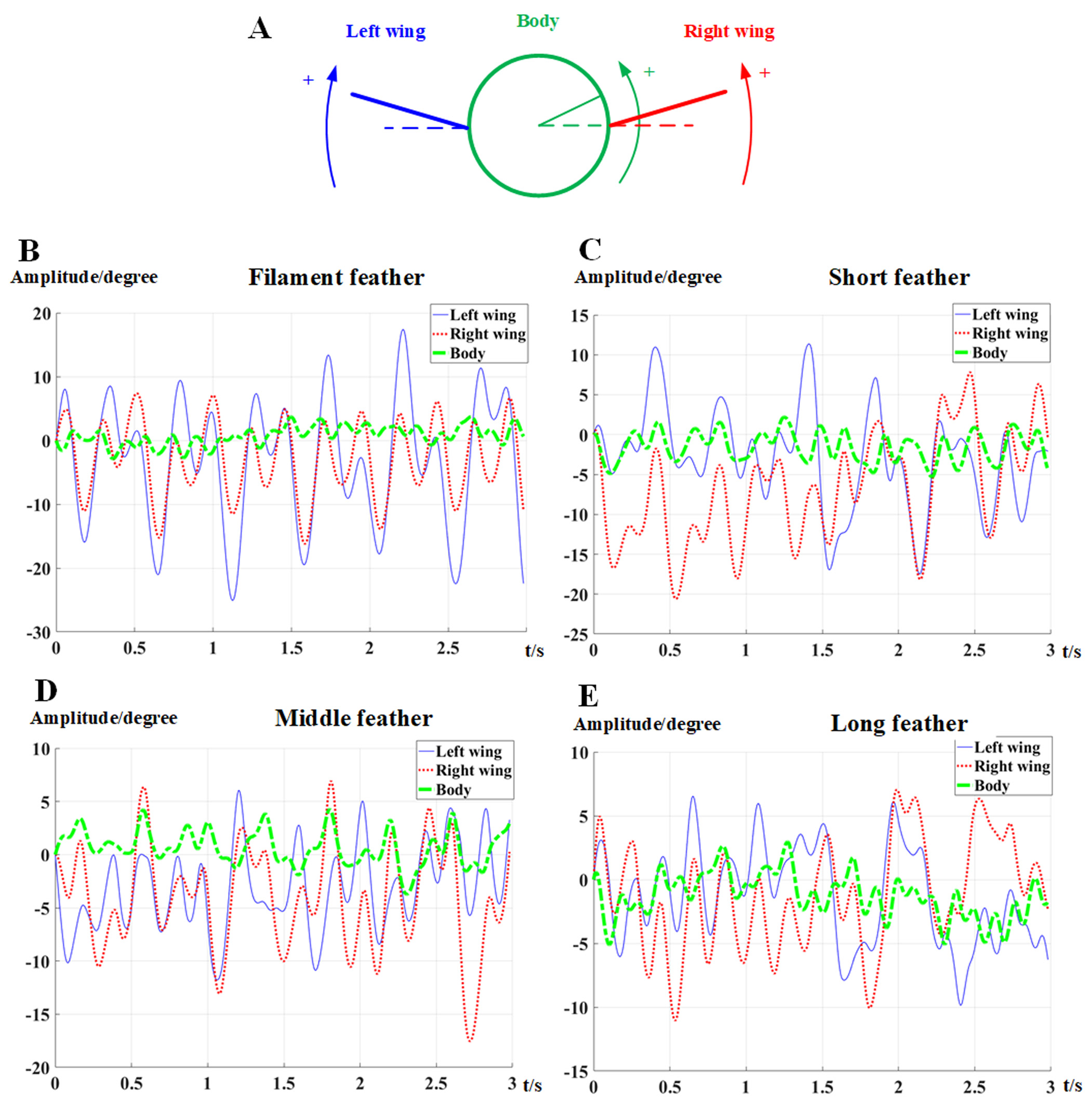

Supplement: S4 Fig — (A) Definition of the flapping angles of the wings. The clockwise rotation is the positive direction for the left wing while the anticlockwise rotation is the positive direction for the right wing. (B) Response of the filament feathers. The largest flapping angle of the wings is around 25°. (C) Response of the short feathers. The largest flapping angle of the wings is about 20°. (D) Response of the middle-sized feathers. The largest flapping angle of the wings is less than 15°. (E) Response of the longest feathers. The largest flapping angle of the wings is less than 10°. This results show that two wings will move up and down simultaneously, which is the flapping motion when the ostrich runs. Longer feather will have smaller flapping angle because of the air resistance during this passive experiment. (TIF) [file pcbi.1006846.s004.tif]

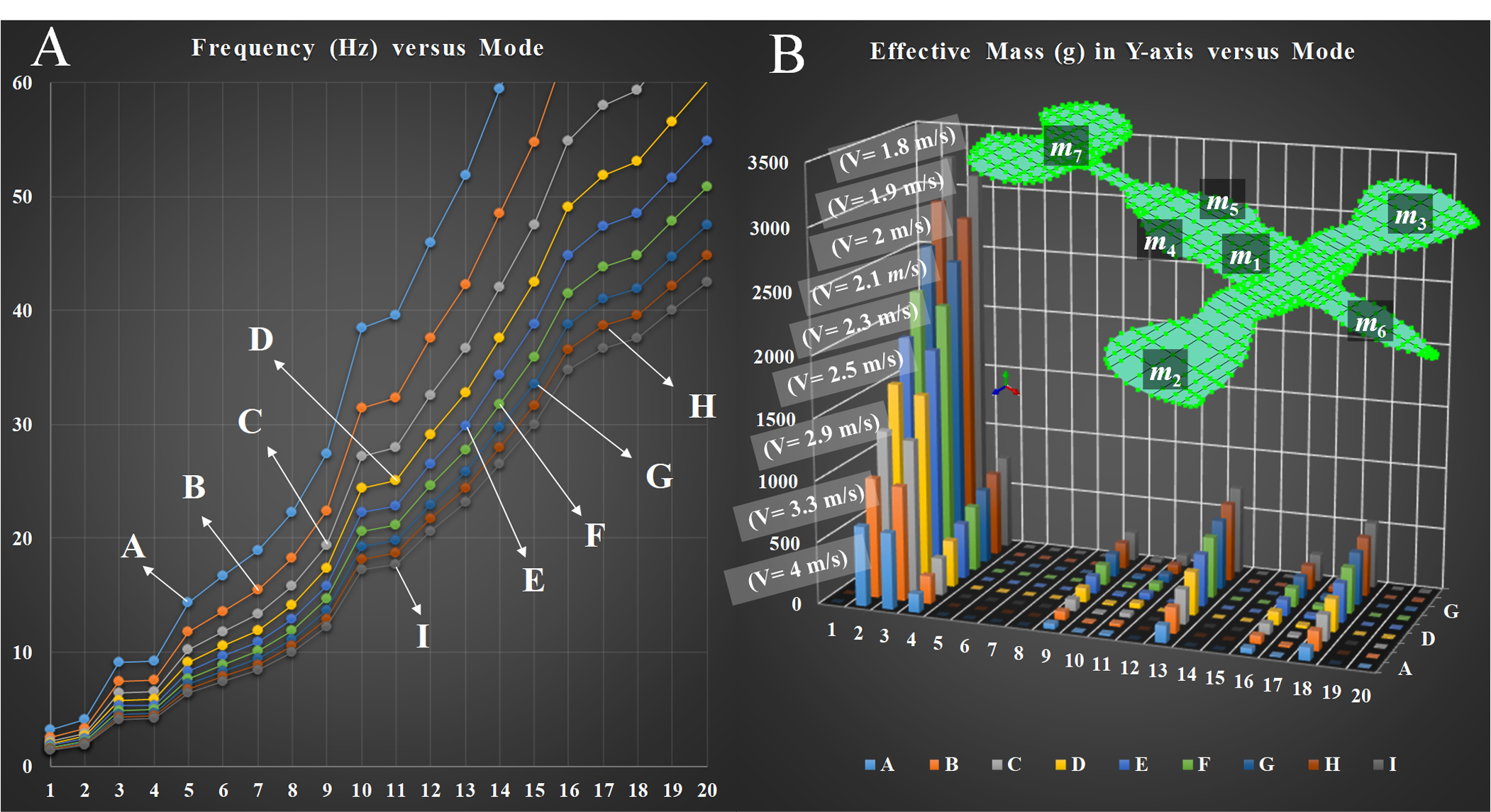

Supplement: S5 Fig — (A) changes of natural frequencies with respect to the modes. (B) effective masses in Y-axis versus modes and velocities to reach to the flapping or second modes. The natural frequency decreases from 4 Hz in mass model A to 1.8 Hz in mass model I in the second mode, hence, as the weight of the creature increases, the velocity in order to reach to the flapping mode might be decreased. (TIF) [file pcbi.1006846.s005.tif]

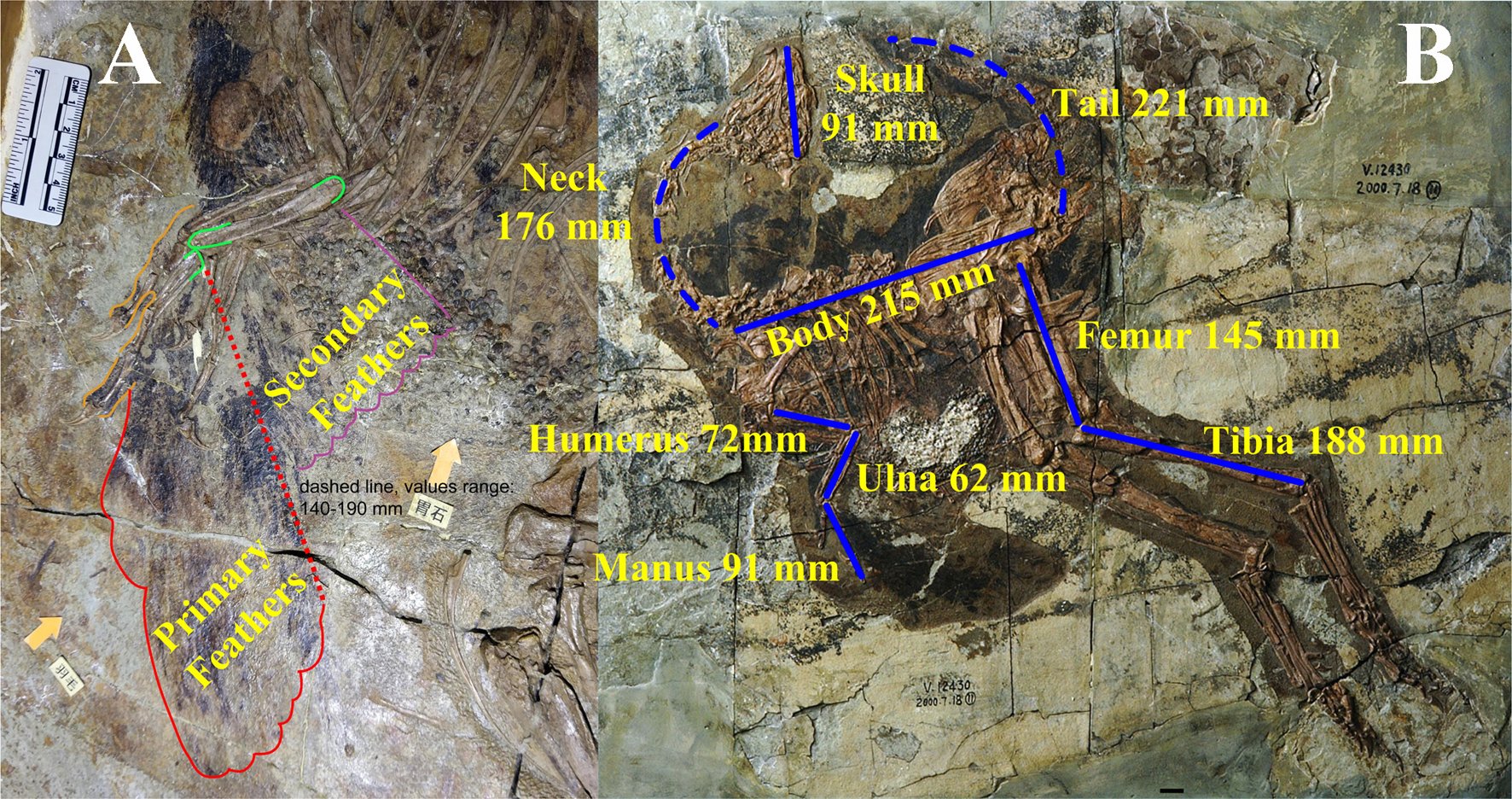

Supplement: S6 Fig — (A) Caudipteryx dongi IVPP V12344 and (B) Caudipteryx sp. IVPP V12430. (TIF) [file pcbi.1006846.s006.tif]
