# Supplementary material for: Identification of avian flapping motion from non-volant winged dinosaurs based on modal effective mass analysis
Source: PLoS Comput Biol. 2019 May 2;15(5):e1006846. doi: 10.1371/journal.pcbi.1006846 (PMC6497222; doi:10.1371/journal.pcbi.1006846)
Supplement: S1 Text — (PDF) [file pcbi.1006846.s011.pdf]

Supplementary Materials

Identification of avian flapping motion from non-volant winged dinosaurs based on modal effective mass analysis

Yaser Saffar Talori[1], Jing-Shan Zhao[1*], Yun-Fei Liu[1], Wen-Xiu Lu[1], Zhi-Heng Li[2] and Jingmai Kathleen O'Connor[2]

*Jing-Shan Zhao
Email: jingshanzhao@mail.tsinghua.edu.cn

This file includes:

Materials and Methods
Vibration Theory
Modal Effective Mass Theory
Interval Analysis
Caudipteryx velocity
References

Materials and Methods

We quantified the fountain of flapping mathematically and in practice. We analyzed a flightless winged dinosaur (*Caudipteryx*) during running and evaluated how this theropod could sense flapping on its wings. Hence, we could obtain invaluable results in the process of beginning of learning flapping using the method of modal effective mass mathematically, FEM simulation and experiments on a reconstructed robot of *Caudipteryx*.

Modal effective mass. To find which mode is most easily excited when the *Caudipteryx* ran on the ground, modal effective mass model was established and validated via finite element method. Modes with relatively high effective masses are readily excited by the running excitation. The *Caudipteryx* was divided into seven lumped mass points, including the main body, two wings, two legs, tail and neck. The seven degree-of-freedom of *Caudipteryx* mechanism based on the reconstruction from the fossil was then used to calculate and simulation with continuous *Caudipteryx* model at computer was accomplished. Both results demonstrated that rolling and flapping modes were easily exited when the *Caudipteryx* ran on the ground.

Reconstruction of *Caudipteryx*. The measurements from *Caudipteryx zoui* BPM 0001 have been used to appraise the whole body of *Caudipteryx* and characterize the appropriate relationship to use for the robot and the mathematical models of this dinosaur. Every part of the robot was fabricated with 3-D printer, guided by information from the fossil.

Experiment and data analysis. First experiment via the reconstructed *Caudipteryx* robot on the testrig were executed and we could obtain the velocity related to the most obvious flapping mode. Second experiment with the reconstructed *Caudipteryx* wings were executed on the ostrich. We fabricated the *Caudipteryx* wings with different size and area. The accelerometer and force sensors were attached to the wings to measure the body and wings rolling angles and lift force during passive flapping. By collecting the data on the testrig and ostrich, we obtained the pure lifts from different wings.

Vibration Theory

The theory of vibration [1] deals with the oscillatory motions of bodies and the forces associated with them. All bodies possessing mass and elasticity are capable of vibration. Thus, most engineering machines and structures experience vibration to some degree, and their design generally requires consideration of their oscillatory behavior. Oscillatory systems can be broadly characterized as linear or nonlinear. For linear systems, the principle of superposition holds, and the mathematical techniques available for their treatment are well developed. In contrast, techniques for the analysis of nonlinear systems are less well known, and difficult to apply. However, some knowledge of nonlinear systems is desirable, because all systems tend to become nonlinear with increasing amplitude of oscillation. There are two general classes of vibrations, free and forced. Free vibration takes place when a system oscillates under the action of forces inherent in the system itself, and when external impressed forces are absent. The system under free vibration will vibrate at one or more of its natural frequencies, which are properties of the dynamical system established by its mass and stiffness distribution. Vibration that takes place under the excitation of external forces is called forced vibration. When the excitation is oscillatory, the system is forced to vibrate at the excitation frequency. If the frequency of excitation coincides with one of the natural frequencies of the system, a condition of resonance is encountered, and dangerously large oscillations may result. Thus, the calculation of the natural frequencies is of major importance in the study of vibrations.

Vibrating systems are all subject to damping to some degree because energy is dissipated by friction and damping. If the damping is small, it has very little influence on the natural frequencies of the system, and hence the calculations for the natural frequencies are generally made on the basis of no damping. On the other hand, damping is of great importance in limiting the amplitude of oscillation at resonance. The number of independent coordinates required to describe the motion of a system is called degrees of freedom of the system. Thus, a free particle undergoing general motion in space will have three degrees of freedom, and a rigid body will have six degrees of freedom, i.e., three components of position and three angles defining its orientation. Furthermore, a continuous elastic body will require an infinite number of coordinates (three for each point on the body) to describe its motion; hence, its degrees of freedom must be infinite. However, in many cases, parts of such bodies may be assumed to be rigid, and the system may be considered to be dynamically equivalent to one having finite degrees of freedom. In fact, a surprisingly large number of vibration problems can be treated with sufficient accuracy by reducing the system to one having a few degrees of freedom.

All systems possessing mass and elasticity are capable of free vibration, or vibration that takes place in the absence of external excitation. Of primary interest for such a system is its natural frequency of vibration. Our objectives here are to learn to write its equation of motion and evaluate its natural frequency, which is mainly a function of the mass and stiffness of the system.

Damping in moderate amounts has little influence on the natural frequency and may be neglected in its calculation. The system can then be considered to be conservative, and the principle of conservation of energy offers another approach to the calculation of the natural

frequency. The effect of damping is mainly evident in the diminishing of the vibration amplitude with time.

The basic vibration model of a simple oscillatory system consists of a mass, a massless spring, and a damper. The mass is considered to be lumped and measured in the SI system as kilograms. The spring supporting the mass is assumed to be of negligible mass. Its force-deflection relationship is considered to be linear, following Hooke's law, $\boldsymbol{F} = \boldsymbol{kx}$, where the stiffness $\boldsymbol{k}$ is measured in newtons/meter and $\boldsymbol{x}$ is the deformation measured in meters. The viscous damping, generally represented by a dashpot, is described by a force proportional to the velocity, or $\boldsymbol{F} = \boldsymbol{c\dot{x}}$ where $\dot{\boldsymbol{x}}$ represents the velocity. The damping coefficient $\boldsymbol{c}$ is measured in newtons/meter/second.

Figure A (left) shows a simple undamped spring-mass system, which is assumed to move only along the vertical direction. It has 1 degree of freedom (DOF), because its motion is described by a single coordinate $\boldsymbol{x}$. When placed into motion, oscillation will take place at the natural frequency $\boldsymbol{f_n}$, which is a property of the system. We now examine some of the basic concepts associated with the free vibration of systems with one degree of freedom. Newton's second law is the first basis for examining the motion of the system. As shown in Figure A (left) the deformation of the spring in the static equilibrium position is Δ, and the spring force $\boldsymbol{k}\Delta$ is equal to the gravitational force w acting on mass m:

$$\boldsymbol{k}\Delta = \boldsymbol{w} = \boldsymbol{mg} \tag{S1}$$

where $\boldsymbol{g}$ is the gravitational coefficient on the earth. By measuring the displacement $\boldsymbol{x}$ from the static equilibrium position, the forces acting on $\boldsymbol{m}$ are $\boldsymbol{k}(\Delta + \boldsymbol{x})$, $\boldsymbol{c\dot{x}}$ and $\boldsymbol{w}$ shown in Figure A (right). With x chosen to be positive in the downward direction, all quantities, force, velocity, and acceleration, are also positive in the downward direction. We now apply Newton's second law of motion to the mass m:

$$\boldsymbol{m\ddot{x}} = \sum \boldsymbol{F} = \boldsymbol{w} - \boldsymbol{k}(\Delta + \boldsymbol{x}) - \boldsymbol{c\dot{x}} \tag{S2}$$

and because $\boldsymbol{k}\Delta = \boldsymbol{w}$, we obtain

$$\boldsymbol{m\ddot{x}} = -\boldsymbol{kx} - \boldsymbol{c\dot{x}} \tag{S3}$$

where $\boldsymbol{c}$ is the damping coefficient.

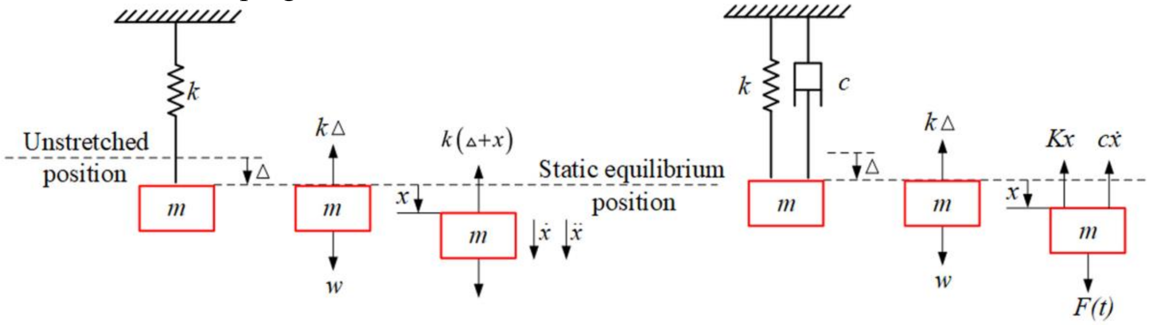

Figure A. Spring, damper and mass system and free-body diagrams.

Equation (S3) can be written as below when $\boldsymbol{c}$ is neglected:

$$\ddot{\boldsymbol{x}} + \omega_n^2 \boldsymbol{x} = 0 \tag{S4}$$

where $\omega_n^2 = \dfrac{\boldsymbol{k}}{\boldsymbol{m}}$ and we conclude by comparison with equation $\ddot{\boldsymbol{x}} = -\omega^2 \boldsymbol{x}$ that the motion is harmonic. A homogeneous second-order linear differential equation (S4) has the following general solution:

$$\boldsymbol{x} = A \sin \omega_n t + B \cos \omega_n t \tag{S5}$$

where *A* and *B* are the two necessary constants that are evaluated from initial conditions $\boldsymbol{x}(0)$ and $\dot{\boldsymbol{x}}(0)$. So equation (S5) can be reduced to

$$\boldsymbol{x} = \frac{\dot{\boldsymbol{x}}(0)}{\omega_n} \sin \omega_n t + \boldsymbol{x}(0) \cos \omega_n t \tag{S6}$$

The natural period of the oscillation is established from $\omega_n \tau = 2\pi$, or

$$\tau = 2\pi \sqrt{\frac{\boldsymbol{m}}{\boldsymbol{k}}} \tag{S7}$$

and the natural frequency is

$$\boldsymbol{f}_n = \frac{1}{\tau} = \frac{1}{2\pi} \sqrt{\frac{\boldsymbol{k}}{\boldsymbol{m}}} \tag{S8}$$

These quantities can be expressed in terms of the static deflection Δ by observing equation (S1), $\boldsymbol{k}\Delta = \boldsymbol{mg}$. Thus, equation (S9) can be expressed in terms of the statical deflection Δ as

$$\boldsymbol{f}_n = \frac{1}{2\pi} \sqrt{\frac{\boldsymbol{g}}{\Delta}} \tag{S9}$$

Note that τ, $\boldsymbol{f}_n$ and ω_n depend only on the mass and stiffness of the system, which are properties of the system. Equation (S3) can be written as (S10) when the damping coefficient, ***c***, is taken into account. From the free-body diagram (Figure A), the dynamics equation is

$$\boldsymbol{m}\ddot{\boldsymbol{x}} + \boldsymbol{kx} + \boldsymbol{c}\dot{\boldsymbol{x}} = \boldsymbol{F}(\boldsymbol{t}) \tag{S10}$$

where $\boldsymbol{F}(\boldsymbol{t})$ is the external force. When $\boldsymbol{F}(\boldsymbol{t}) = 0$ in equation (S10), we have the homogeneous differential equation whose solution corresponds physically to that of free-damped vibration. When $\boldsymbol{F}(\boldsymbol{t}) \neq 0$, we obtain the particular solution that is due to the excitation irrespective of the homogeneous solution.

With the homogeneous equation

$$m\ddot{x}+c\dot{x}+kx=0 \tag{S11}$$

the traditional approach is to assume a solution of the form

$$x=e^{st} \tag{S12}$$

where s is a constant. Upon substitution into the differential equation, we obtain

$$(ms^2+cs+k)e^{st}=0 \tag{S13}$$

which is satisfied for all values of t when

$$s^2+\frac{c}{m}s+\frac{k}{m}=0 \tag{S14}$$

Equation (S14), which is known as the characteristic equation, has two roots, real or complex:

$$s_{1,2}=-\frac{c}{2m}\pm\sqrt{\left(\frac{c}{2m}\right)^2-\frac{k}{m}} \tag{S15}$$

Hence, the general solution is given by the equation

$$x=Ae^{s_1t}+Be^{s_2t} \tag{S16}$$

where A and B are constants to be evaluated from the initial conditions $x(0)$ and $\dot{x}(0)$.

Substituting equation (S15) into (S16) gives

$$x=e^{-(c/2m)t}\left(Ae^{\left(\sqrt{(c/2m)^2-k/m}\right)t}+Be^{-\left(\sqrt{(c/2m)^2-k/m}\right)t}\right) \tag{S17}$$

The first term, $e^{-(c/2m)t}$, is an exponentially decaying function of time. The behavior of the terms in the parentheses, however, depends on whether the numerical value within the radical is positive, zero, or negative.

When the damping term $(c/2m)^2$ is larger than *k*/*m*, the exponents in the previous equation are real numbers and no oscillations are possible. We refer to this case as overdamped.

When the damping term $(c/2m)^2$ is less than *k*/*m*, the exponent becomes an imaginary number, $\pm i\left(\sqrt{k/m-(c/2m)^2}\right)t$. Because

$$e^{\pm i\left(\sqrt{k/m-(c/2m)^2}\right)t}=\cos\sqrt{\frac{k}{m}-\left(\frac{c}{2m}\right)^2}\,t\pm i\sin\sqrt{\frac{k}{m}-\left(\frac{c}{2m}\right)^2}\,t \tag{S18}$$

the terms of equation (S18) within the parentheses are oscillatory. We refer to this case as underdamped.

In the limiting case between the oscillatory and nonoscillatory motion, $(\boldsymbol{c}/2\boldsymbol{m})^2 = \boldsymbol{k}/\boldsymbol{m}$, and the radical is zero. The damping corresponding to this case is called critical damping, $\boldsymbol{c}_c$.

$$\boldsymbol{c}_c = 2\boldsymbol{m}\sqrt{\frac{\boldsymbol{k}}{\boldsymbol{m}}} = 2\boldsymbol{m}\omega_n = 2\sqrt{\boldsymbol{km}} \quad \text{(S19)}$$

Any damping can then be expressed in terms of the critical damping by a non-dimensional number, ξ, called the damping ratio:

$$\xi = \frac{\boldsymbol{c}}{\boldsymbol{c}_c} \quad \text{(S20)}$$

where $\boldsymbol{c}_c$ is the critical damping coefficient, and we can also express $\boldsymbol{s}_{1,2}$ in terms of ξ as follows:

$$\frac{\boldsymbol{c}}{2\boldsymbol{m}} = \xi\left(\frac{\boldsymbol{c}_c}{2\boldsymbol{m}}\right) = \xi\omega_n \quad \text{(S21)}$$

Equation (S15) then becomes

$$\boldsymbol{s}_{1,2} = \left(-\xi \pm \sqrt{\xi^2 - 1}\right)\omega_n \quad \text{(S22)}$$

The three cases of damping discussed here now depend on whether ξ is greater than, less than, or equal to unity. Furthermore, the differential equation of motion can now be expressed in terms of ξ and ω_n as

$$\ddot{\boldsymbol{x}} + 2\xi\omega_n\dot{\boldsymbol{x}} + \omega_n^2\boldsymbol{x} = \frac{1}{\boldsymbol{m}}\boldsymbol{F}(\boldsymbol{t}) \quad \text{(S23)}$$

This form of the equation for single-DOF systems will be found to be helpful in identifying the natural frequency and the damping of the system. We will frequently encounter this equation in the modal summation for multi-DOF systems.

Figure B shows equation (S22) plotted in a complex plane with ξ along the horizontal axis. When $\xi = 0$, equation (S22) reduces to $\boldsymbol{s}_{1,2}/\omega = \pm\boldsymbol{i}$ so that the roots on the imaginary axis correspond to the undamped case. For $0 \le \xi \le 1$, equation (S22) can be rewritten as

$$\frac{\boldsymbol{s}_{1,2}}{\omega_n} = -\xi \pm \boldsymbol{i}\sqrt{1-\xi^2} \quad \text{(S24)}$$

The roots s_1 and s_2 are then conjugate complex points on a circular that are converging at the point $s_{1,2} / \omega_n = -1.0$. As ξ increases beyond unity, the roots separate along the horizontal axis and remain real numbers. With this diagram in mind, we are now ready to examine the solution given by equation (S17).

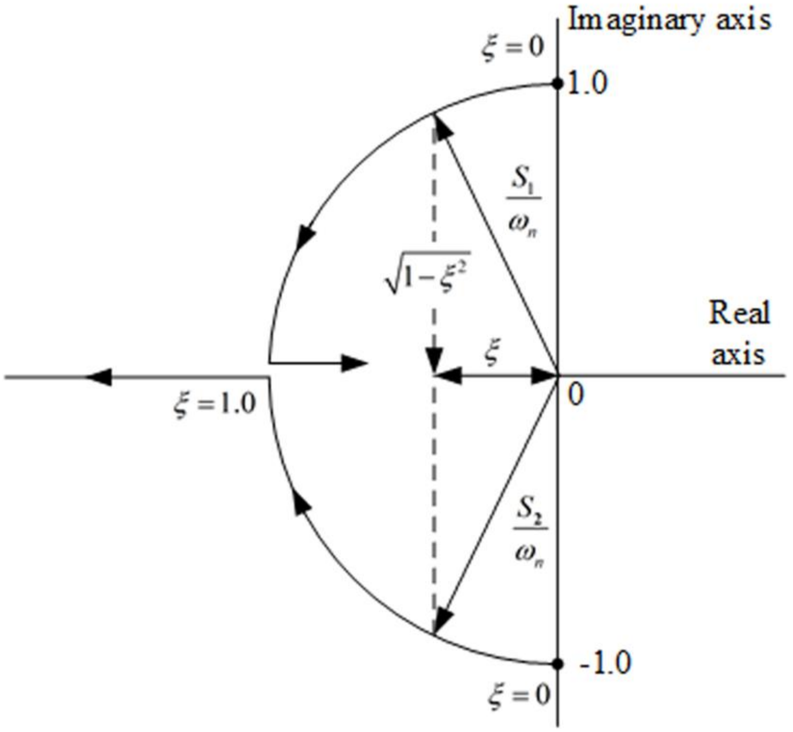

Figure B. Equation (S22) plotted in a complex plane with ξ along the horizontal axis.

Oscillatory motion [$\xi < 1.0$ (Underdamped Case)]. By substituting equation (S22) into (S17), the general solution becomes

$$x = e^{-\xi \omega_n t} \left(A e^{i \sqrt{1-\xi^2} \omega_n t} + B e^{-i \sqrt{1-\xi^2} \omega_n t} \right) \tag{S25}$$

This equation can also be written in either of the following two forms:

$$x = X e^{-\xi \omega_n t} \sin \left(\sqrt{1-\xi^2} \omega_n t + \phi \right) \tag{S26}$$

$$x = e^{-\xi \omega_n t} \left(C_1 \sin \sqrt{1-\xi^2} \omega_n t + C_2 \cos \sqrt{1-\xi^2} \omega_n t \right) \tag{S27}$$

where the arbitrary constants X, ϕ, or C_1, C_2 are determined from initial conditions. With initial conditions $x(0)$ and $\dot{x}(0)$, equation (S27) can be shown to reduce to

$$x = e^{-\xi \omega_n t} \left(\frac{\dot{x}(0) + \xi \omega_n x(0)}{\omega_n \sqrt{1-\xi^2}} \sin \sqrt{1-\xi^2} \omega_n t + x(0) \cos \sqrt{1-\xi^2} \omega_n t \right) \tag{S28}$$

The equation indicates that the frequency of damped oscillation is equal to

$$\omega_d = \frac{2\pi}{\tau_d} = \omega_n\sqrt{1-\xi^2} \tag{S29}$$

Therefore, the natural frequency of the oscillation considering damping coefficient is a function of ω_n (free natural frequency without considering damping in the system). As it is shown in the general nature of the oscillatory motion in Figure C, a system can vibrate by its natural frequency but using damper to decrease the amplitude of oscillation to fully damp the whole system. The damping ratio of a vibrating system is depending on the damping coefficient. Hence, small value of damping almost cannot effect on free natural vibration and it only reduces the amplitude of vibration in long term.

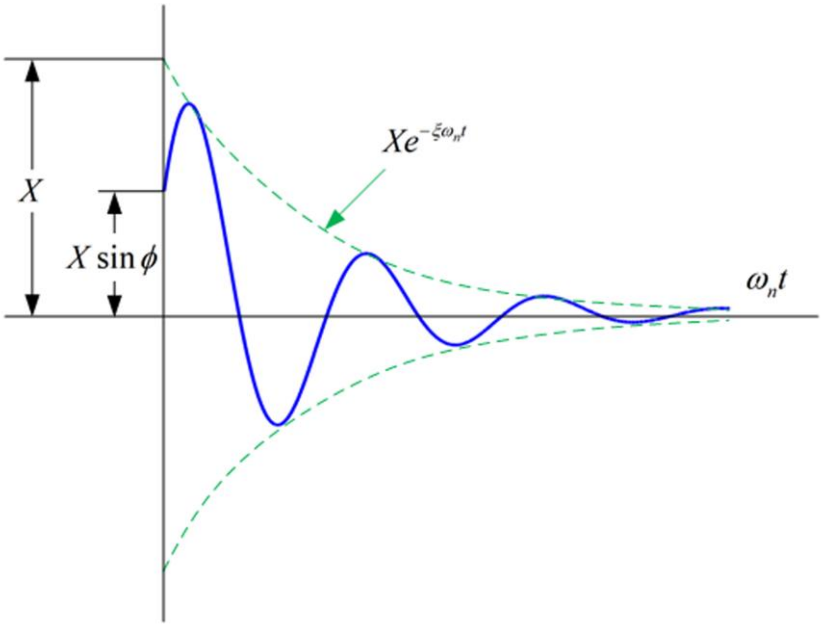

Figure C. The general nature of oscillatory motion (Damped oscillation, $\xi < 1.0$).

Natural Frequencies and Mode Shapes. To calculate the natural frequencies and mode shapes for multiple degree-of-freedom (DOF) rigid-body systems, we first linearize the equations of motion (EOM) and express them in the matrix form:

$$[\mathbf{M}]\{\ddot{\boldsymbol{x}}\} + [\mathbf{C}]\{\dot{\boldsymbol{x}}\} + [\mathbf{K}]\{\boldsymbol{x}\} = \{\boldsymbol{F(t)}\} \tag{S30}$$

Here, [**M**], [**C**]and [**K**] represent the system mass, damping, and stiffness matrices, the vector $\{\boldsymbol{x}\}$ represents the changes in all the generalized coordinates from their equilibrium values, and the vector $\{\boldsymbol{F}\}$ represents all the forces acting on the system. Then, we set the damping matrix and force vector to zero to get free, undamped response.

$$[\mathbf{M}]\{\ddot{\boldsymbol{x}}\} + [\mathbf{K}]\{\boldsymbol{x}\} = \{\mathbf{0}\} \tag{S31}$$

Following the pattern for single DOF systems, we look for a solution to this equation of the form $\{\boldsymbol{x}\} = e^{st}\{\boldsymbol{u}\}$ where $\boldsymbol{s} = \boldsymbol{j}\omega$.

This equation describes a steady-state, undamped solution, with representing the mode shape of the oscillations. Substituting this into the differential EOM gives

$$([\mathbf{K}]-\omega^2[\mathbf{M}])\{\boldsymbol{u}\}=\{\mathbf{0}\} \qquad \text{(S32)}$$

The problem now is to find ω^2 and $\{\boldsymbol{u}\}$ that satisfy this algebraic equation. This is called an eigenvalue problem. For this equation to have a non-zero solution for a nonzero vector $\{\boldsymbol{u}\}$, we require the determinant of the coefficient matrix to be zero.

$$\det([\mathbf{K}]-\omega^2[\mathbf{M}])=0 \qquad \text{(S33)}$$

If the matrices [**M**] and [**K**] are N×N matrices, the solution to this equation yields N values for ω^2, and hence N values for ω. These are N natural frequencies of the system. Associated with each of these frequencies is a mode shape $\{\boldsymbol{u}\}$. The ω^2 is called the eigenvalue of the system, and the vector $\{\boldsymbol{u}\}$ is called the eigenvector of the system.

Modal Effective Mass Theory

One of the most important tasks in analysis and modal survey planning is the selection of target modes. The target modes are those mode shapes that are determined to be dynamically important using some definition. While there are many measures of dynamic importance [2-8], one of the measures that is of greatest interest to a structural dynamicist, is the contribution of each mode shape to the dynamic loads at an interface. The interface can represent a connection to ground, a connection of the substructure to the rest of a system, or locations where external forces are applied. Dynamically important modes contribute significantly to the interface loads and must be retained in any reduced analytical representation of the substructure. Therefore, these modes must be identified during a ground vibration.

Modal participation factors have been widely used by the civil engineers to determine the modal response of buildings to ground motion from earthquakes, while structural dynamicists have extensively used *Effective Mass* [9] to determine dynamic importance. The advantage of *Effective Mass* is that it is an absolute measure of dynamic importance in contrast to other measures that only indicate the importance of one mode shape with respect to another. The advantage of an absolute measure is that the original modal representation of the FEM can be truncated such that a desired fraction of the total *Effective Mass* is retained, guaranteeing that the reduced representation of the substructure will be accurate. The usual dynamic analysis techniques express structure deformation in a new base of rigid modes and elastic modes. Each mode is characterized by a modal mass and effective masses associated with different directions. The normalization of these modes is arbitrary, this implies that they have no physical meaning when considered alone. On the other hand, the effective mass has a physical meaning and allows, in a lot of cases, simplification of the computation of deformations and stresses. They allow a trade-off of useful modes and simple computations on complex structures. Indeed, each mode can be interpreted as a mass-spring-damper system oriented in the rigid modes space along a specific direction. The mass in this case is the effective mass. When a force is applied on the internal degrees of freedom, each elastic mode will be excited by the projection of the force on this mode. Moreover, the reaction at the junction DOF implied by a mode will have a direction parallel to the mode specific direction [10-15].

The effective mass $\boldsymbol{m}_{eff,k}$ of mode k is the fraction of the total static mass that can be attributed to this mode (static inertia for rotation modes). The vectors define a set of directions in the rigid mode space (a 6-dimension space in general). Each effective mode can be represented by a mass-spring-dashpot system oriented along vector in the rigid mode space. The mass value is $\boldsymbol{m}_{eff,k}$, the spring constant is $\boldsymbol{k}_{eff,k}$ and the damping coefficient is unchanged. The elastic behavior of the structure can be represented in the rigid mode space by this set of 1-D mass-spring-dashpot systems (Figure D) [10-15].

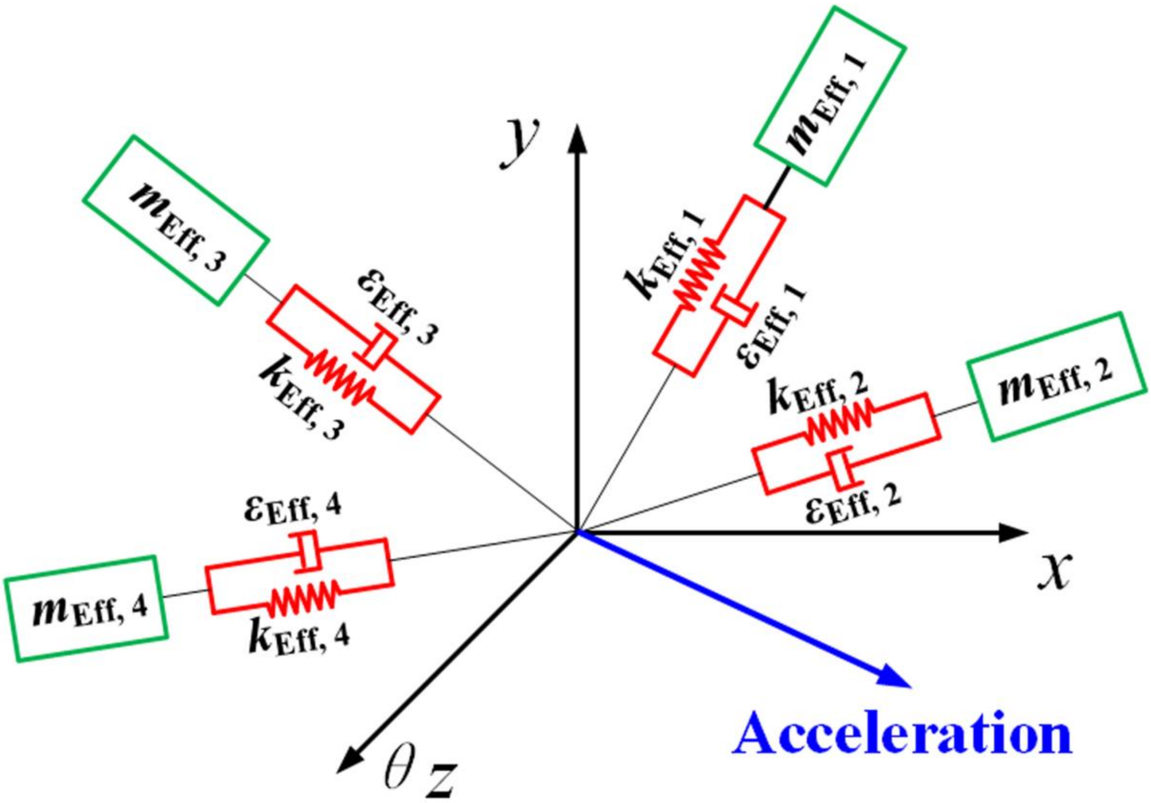

Figure D. Representation of elastic modes in rigid modes space (X, Y, Θz). (A general representation of a structure by a set of 1-D mass-spring-dashpot systems in the space of rigid modes). The effective masses distribution can easily characterize the relative contribution of the modes when the structure is excited along a direction with a given frequency [10].

For brevity, the modal effective mass provides a method for judging the "significance" of a vibration mode. Modes with relatively high effective masses can be readily excited by base excitation. On the other hand, modes with low effective masses cannot be readily excited in this manner. Consider a modal transient or frequency response function analysis via the finite element method. Also consider that the system is a multi-degree-of-freedom system. Only a limited number of modes should be included in the analysis. The number should be enough so that the total effective modal mass of the model is at least 90% of the actual mass (By Tom Irvine) [16-21].

The modal effective mass is a modal dynamic property of a structure associated with the modal characteristics; natural frequencies, mode shapes, generalized masses, and participation factors. The modal effective mass is a measure to classify the importance of a mode shape when a structure is excited by base acceleration (enforced acceleration). A high effective mass will lead to a high reaction force at the base, while mode shapes with low associated modal effective mass are barely excited by base acceleration and will give low reaction forces at the base. The effect of local modes is not well described with modal effective masses (Shunmugavel 1995, Witting 1996). The modal effective mass matrix is a 6×6 mass matrix. Within this matrix the coupling between translations and rotations, for

a certain mode shape, can be traced. The summation over all modal effective masses will result in the mass matrix as a rigid-body [22-26].

A Single Degree of Freedom (SDOF) system with a discrete mass *m*, a damper element *c* and a spring element *k* is placed on a moving base that is accelerated with an acceleration $\ddot{u}(t)$. The resulting displacement of the mass is $x(t)$, the natural (circular) frequency $\omega_n = \sqrt{\frac{k}{m}}$, the critical damping constant $c_{crit} = 2\sqrt{km}$ and the damping ratio $\xi = \frac{c}{c_{crit}}$ are introduced. The amplification factor is defined as $Q = \frac{1}{2\xi}$.

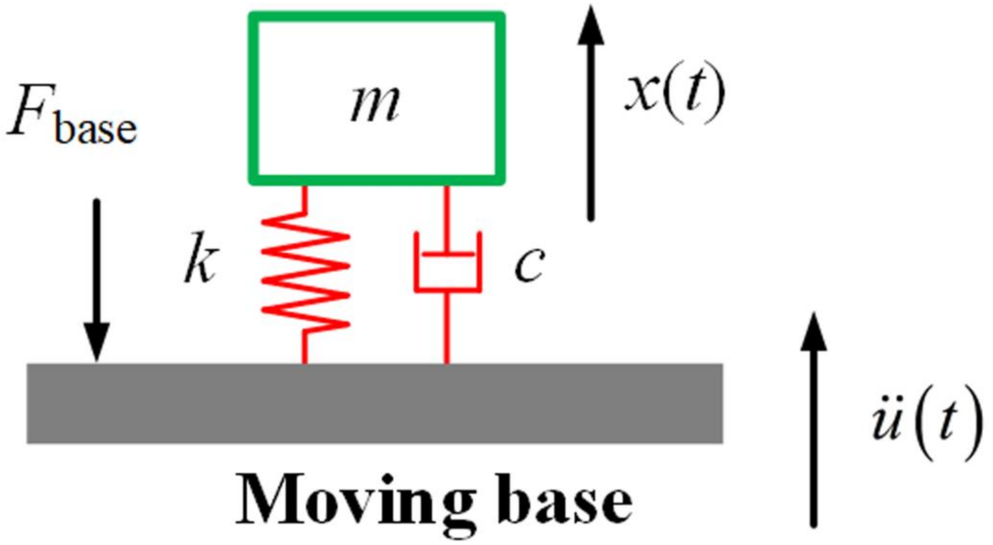

Figure E. Enforced acceleration of a damped SDOF system [22].

A relative motion $z(t)$ is introduced, which is the displacement of the mass with respect to the base. The relative displacement is $z(t) = x(t) - u(t)$. The equation for the relative motion is $\ddot{z}(t) + 2\xi\omega_n\dot{z}(t) + \omega_n^2 z(t) = -\ddot{u}(t)$. The enforced acceleration of the SDOF system is transformed into an external force. The absolute displacement $x(t)$ can be calculated from $\ddot{x}(t) = \ddot{z}(t) + \ddot{u}(t) = -2\xi\omega_n\dot{z}(t) - \omega_n^2 z(t)$. The reaction force $F_{base}(t)$, due to the enforced acceleration $\ddot{u}(t)$, is a summation of the spring force and the damping force $F_{base}(t) = kz(t) + c\dot{z}(t) = -m\{\ddot{z}(t) + \ddot{u}(t)\} = -m\ddot{x}(t)$. Assuming harmonic vibration, we can write the enforced acceleration $\ddot{u}(t) = \ddot{U}(\omega)e^{j\omega t}$ and also the relative motion $z(t)$

$z(t) = Z(\omega)e^{j\omega t}$, $\dot{z}(t) = j\omega Z(\omega)e^{j\omega t}$ and $\ddot{z}(t) = -\omega^2 Z(\omega)e^{j\omega t}$. Therefore, the absolute acceleration of the SDOF dynamic system is

$$\ddot{x}(t) = \ddot{X}(\omega)e^{j\omega t} = -\omega^2 X(\omega)e^{j\omega t} \tag{S34}$$

Equation $\ddot{z}(t) + 2\xi\omega_n\dot{z}(t) + \omega_n^2 z(t) = -\ddot{u}(t)$ can be transformed into the frequency domain

$$[-\omega^2 + 2j\xi\omega_n\omega + \omega_n^2]Z(\omega) = -\ddot{U}(\omega) \tag{S35}$$

We are able to express the relative displacement $z(\omega)$ in the enforced acceleration $\ddot{U}(\omega) = -\omega^2 U(\omega)$,

$Z(\omega) = \left(\frac{\omega}{\omega_n}\right)^2 H\left(\frac{\omega}{\omega_n}\right)U(\omega)$ where $H(\omega) = \dfrac{1}{1-\left(\frac{\omega}{\omega_n}\right)^2 + 2j\xi\left(\frac{\omega}{\omega_n}\right)}$ is the frequency response function. Using equation $\ddot{x}(t) = \ddot{z}(t) + \ddot{u}(t) = -2\xi\omega_n\dot{z}(t) - \omega_n^2 z(t)$, we can write the absolute acceleration $\ddot{X}(\omega)$ as

$$\ddot{X}(\omega) = -\omega^2[Z(\omega) + U(\omega)] = -\omega^2\left[1 + \left(\frac{\omega}{\omega_n}\right)^2 H\left(\frac{\omega}{\omega_n}\right)\right]U(\omega) \tag{S35}$$

or

$$\ddot{X}(\omega) = \left[1 + \left(\frac{\omega}{\omega_n}\right)^2 H\left(\frac{\omega}{\omega_n}\right)\right]\ddot{U}(\omega) \tag{S36}$$

With the aid of equation $F_{base}(t) = kz(t) + c\dot{z}(t) = -m\{\ddot{z}(t) + \ddot{u}(t)\} = -m\ddot{x}(t)$, the reaction force at the base $F_{base}(\omega)$ now becomes

$$F_{base}(\omega) = m\ddot{X}(\omega) = m\left[1 + \left(\frac{\omega}{\omega_n}\right)^2 H\left(\frac{\omega}{\omega_n}\right)\right]\ddot{U}(\omega) \tag{S37}$$

In this frame the mass *m* is the effective mass $M_{eff} = m$. The reaction force $F_{base}(\omega)$ is proportional to the effective mass M_{eff} and the base excitation $\ddot{U}(\omega)$ multiplied by the amplification $1 + \left(\frac{\omega}{\omega_n}\right)^2 H\left(\frac{\omega}{\omega_n}\right)$. Similar relations will be derived for multi-degrees of freedom (MDOF) dynamic systems. When the excitation frequency is equal to the natural frequency of the SDOF $\omega = \omega_n$, the reaction force becomes

$$\left|F_{base(\omega_n)}\right| = \left| m\left[1+\frac{1}{2j\xi}\right]\ddot{U}(\omega_n)\right| \approx M_{eff}Q\ddot{U}(\omega_n) \tag{S38}$$

Equation $F_{base}(\omega) = m\ddot{X}(\omega) = m\left[1+\left(\frac{\omega}{\omega_n}\right)^2 H\left(\frac{\omega}{\omega_n}\right)\right]\ddot{U}(\omega)$ can then be written in a dimensionless form

$$\frac{F_{base}(\omega)}{m\ddot{U}(\omega)} = \left[1+\left(\frac{\omega}{\omega_n}\right)^2 H\left(\frac{\omega}{\omega_n}\right)\right] \tag{S39}$$

Modal Effective Masses of an MDOF System. The undamped (matrix) equations of motion for a free elastic body can be written as

$$[M]\{\ddot{x}(t)\}+[K]\{x(t)\}=\{F(t)\} \tag{S40}$$

The external or boundary degrees of freedom are denoted with the index *j* and the internal degrees of freedom with the index *i*. The structure will be excited at the boundary DOFs; 3 translations and 3 rotations.

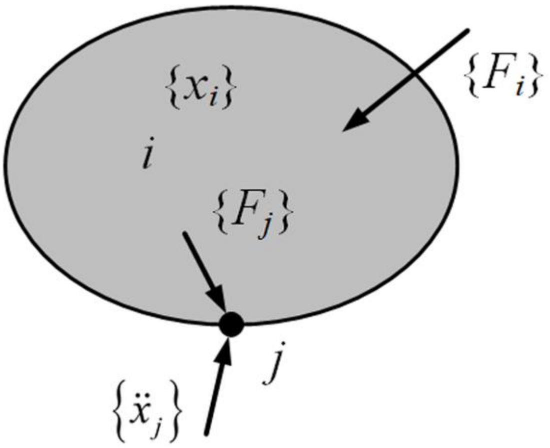

Figure F. Enforced structure [22].

The number of boundary degrees of freedom is less than or equal to 6. The DOFs and forces are illustrated in Figure F. Equation (22) may be partitioned as follows

$$\begin{bmatrix} \mathbf{M}_{ii} & \mathbf{M}_{ij} \\ \mathbf{M}_{ji} & \mathbf{M}_{jj} \end{bmatrix}\begin{Bmatrix} \ddot{x}_i \\ \ddot{x}_j \end{Bmatrix} + \begin{bmatrix} \mathbf{K}_{ii} & \mathbf{K}_{ij} \\ \mathbf{K}_{ji} & \mathbf{K}_{jj} \end{bmatrix}\begin{Bmatrix} x_i \\ x_j \end{Bmatrix} = \begin{Bmatrix} F_i \\ F_j \end{Bmatrix} \tag{S41}$$

It is proposed by Craig to depict the displacement vector $\{x(t)\}$ on a basis of 6-rigid-body modes $[\Phi_r]$ with $\{x_j\}=[I]$ and elastic mode shapes $[\Phi_p]$ with fixed external degrees of freedom $\{x_j\}=[0]$ calculated from the eigenvalue problem $([\mathbf{K}_{ii}]-\lambda_p[\mathbf{M}_{ii}])[\Phi_{ii}]=[0]$. $\{x\}$ can be expressed as [27]

$$\{\boldsymbol{x}\} = [\boldsymbol{\Phi}_r]\{\boldsymbol{x}_j\} + [\boldsymbol{\Phi}_p]\{\boldsymbol{\eta}_p\} = \begin{bmatrix}\boldsymbol{\Phi}_r & \boldsymbol{\Phi}_p\end{bmatrix}\begin{Bmatrix}\boldsymbol{x}_j \\ \boldsymbol{\eta}_p\end{Bmatrix} = [\boldsymbol{\Psi}]\{\boldsymbol{X}\} \tag{S42}$$

The static modes can be obtained, assuming zero inertia effects, and $\{\boldsymbol{F}_j\} = \{\boldsymbol{0}\}$, and prescribe successively a unit displacement for the 6 boundary DOFs, thus $\{\boldsymbol{x}_j\} = [\boldsymbol{I}]$. Equation (S41) may be written as follows

$$\begin{bmatrix}\boldsymbol{K}_{ii} & \boldsymbol{K}_{ij} \\ \boldsymbol{K}_{ji} & \boldsymbol{K}_{jj}\end{bmatrix}\begin{Bmatrix}\boldsymbol{x}_i \\ \boldsymbol{x}_j\end{Bmatrix} = \begin{Bmatrix}0_i \\ 0\end{Bmatrix} \tag{S43}$$

Enforced displacement $\{\boldsymbol{x}_j\}$ will not introduce reaction forces in boundary DOFs. From the first equation of (S43) we find for $\{\boldsymbol{x}_i\}$

$$[\boldsymbol{K}_{ii}]\{\boldsymbol{x}_i\} + [\boldsymbol{K}_{ij}]\{\boldsymbol{x}_j\} = [0] \tag{S44}$$

Hence

$$\{\boldsymbol{x}_i\} = -[\boldsymbol{K}_{ii}]^{-1}[\boldsymbol{K}_{ij}]\{\boldsymbol{x}_j\} \tag{S45}$$

And therefore

$$[\Phi_{ij}] = -[\boldsymbol{K}_{ii}]^{-1}[\boldsymbol{K}_{ij}][\boldsymbol{I}] = -[\boldsymbol{K}_{ii}]^{-1}[\boldsymbol{K}_{ij}] \tag{S46}$$

The static transformation now becomes

$$\{\boldsymbol{x}\} = \begin{Bmatrix}\boldsymbol{x}_i \\ \boldsymbol{x}_j\end{Bmatrix} = \begin{bmatrix}\Phi_{ij} \\ \boldsymbol{I}\end{bmatrix}\{\boldsymbol{x}_j\} = [\Phi_r]\{\boldsymbol{x}_j\} \tag{S47}$$

Using equation (S43) it follows that

$$[\boldsymbol{K}][\Phi_r] = \{0\} \tag{S48}$$

Assuming fixed external degrees of freedom $\{\boldsymbol{x}_j\} = \{0\}$ and also assuming harmonic motions $\boldsymbol{x}(\boldsymbol{t}) = \boldsymbol{X}(\omega)\boldsymbol{e}^{j\omega t}$ the eigenvalue problem can be stated as

$$([\boldsymbol{K}_{ii}] - \lambda_{k,p}[\boldsymbol{M}_{ii}])\{\boldsymbol{X}(\lambda_{k,p})\} = \{0\} \tag{S49}$$

or more generally as

$$([\boldsymbol{K}_{ii}] - \lambda_k[\boldsymbol{M}_{ii}])\{\Phi_{ip}\} = \{0\} \tag{S50}$$

where λ_k is the eigenvalue associated with the mode shape $\{\phi_{ip,k}\}$, $\boldsymbol{k} = 1, 2, \ldots \boldsymbol{j}$. The internal degrees of freedom $\{\boldsymbol{x}_i\}$ will be projected on the set of orthogonal mode shapes (modal matrix) $[\Phi_{ip}]$, thus

$$\{x_i\} = [\Phi_{ip}]\{\eta_p\} \tag{S51}$$

The modal transformation becomes

$$\{x\} = \begin{Bmatrix} x_i \\ x_j \end{Bmatrix} = \begin{bmatrix} \Phi_{ip} \\ 0 \end{bmatrix} \{\eta_p\} = [\Phi_p]\{\eta_p\} \tag{S52}$$

The Craig–Bampton (CB) transformation matrix $[\Psi]$ is

$$\{x\} = \begin{bmatrix} \Phi_r & \Phi_p \end{bmatrix} \begin{Bmatrix} x_j \\ \eta_p \end{Bmatrix} = [\Psi]\{X\} \tag{S53}$$

where $[\Phi_r]$ the rigid body modes, $[\Phi_p]$ the modal matrix, $\{x_j\}$ the external or boundary degrees of freedom ($j \le 6$) and $\{\eta_p\}$ the generalized coordinates. In general, the number of generalized coordinates *P* is much less than the total number of degrees of freedom $n = i + j,\ p \ll i$.

The CB transformation equation (S53) will be substituted into equation (S40) by assuming equal potential and kinetic energies, hence

$$[\Psi]^T[M][\Psi]\{\ddot{x}\} + [\Psi]^T[K][\Psi]\{x\} = [\Psi]^T\{F(t)\} = \{f(t)\} \tag{S54}$$

further elaborated it is found

$$\begin{bmatrix} M_{rr} & M_{jp} \\ M_{pj} & \langle m_p \rangle \end{bmatrix} \begin{Bmatrix} \ddot{x}_j \\ \ddot{\eta}_p \end{Bmatrix} + \begin{bmatrix} \tilde{K}_{jj} & K_{jp} \\ K_{pj} & \langle K_p \rangle \end{bmatrix} \begin{Bmatrix} x_j \\ \eta_p \end{Bmatrix} = \begin{bmatrix} \Phi_{ij} & \Phi_P \\ I & 0 \end{bmatrix}^T \begin{Bmatrix} F_i \\ F_j \end{Bmatrix} \tag{S55}$$

where

$[M_{rr}]$ the 6×6 rigid body mass matrix with respect to the boundary DOFs,

$[\tilde{K}_{jj}]$ the Guyan reduced stiffness matrix (*j*-set),

$\langle m_p \rangle$ the diagonal matrix of generalized masses, $\langle m_p \rangle = [\Phi_p][M][\Phi_p]$,

$\langle K_p \rangle$ the diagonal matrix of generalized stiffnesses,

$\langle K_p \rangle = [\Phi_p]^T K[\Phi_p] = \langle \lambda_p \rangle \langle m_p \rangle = \langle \omega_p^2 \rangle \langle m_p \rangle$,

$[K_{ip}] = [\Phi_{ij}]^T[K_{ii}][\Phi_p] + [K_{ji}][\Phi_p] = (-[K_{ij}]^T[K_{ii}]^{-1}[K_{ii}] + [K_{ji}][\Phi_p]) = [0]$,

$[K_{pi}] = [K_{ip}]^T = [0]$,

$[\tilde{K}_{jj}] = [\Phi_r]^T[K][\Phi_r] = [0]$.

Thus equation (S55) becomes

$$\begin{bmatrix} M_{rr} & L^T \\ L & \langle m_p \rangle \end{bmatrix} \begin{Bmatrix} \ddot{x}_j \\ \ddot{\eta}_p \end{Bmatrix} + \begin{bmatrix} 0 & 0 \\ 0 & \langle m_p \lambda_p \rangle \end{bmatrix} \begin{Bmatrix} x_j \\ \eta_p \end{Bmatrix} = \begin{bmatrix} \Phi_{ij} & \Phi_P \\ I & 0 \end{bmatrix}^T \begin{Bmatrix} 0 \\ F_j \end{Bmatrix} = \begin{Bmatrix} F_j \\ 0 \end{Bmatrix} \tag{S56}$$

where $[M_{jp}] = [\Phi_r]^T [M][\Phi_p] = [L]^T$, $[L]^T$ is the matrix with the modal participation factors, $L_{kl} = \{\phi_{r,k}\}^T [M]\{\phi_{p,l}\}$, $k = 1,2,...,6$, $l = 1,2,..., p$. The matrix of modal participation factors couples the rigid-body modes $[\Phi_r]$ with the elastic modes $[\Phi_p]$ and $\{F_i\} = \{0\}$. No internal loads are applied. Introducing the modal damping ratios ξ_p equation (S56) can be written as follows

$$\begin{bmatrix} M_{rr} & L^T \\ L & \langle m_p \rangle \end{bmatrix} \begin{Bmatrix} \ddot{x}_j \\ \ddot{\eta}_p \end{Bmatrix} + \begin{bmatrix} 0 & 0 \\ 0 & \langle 2 m_p \xi_p \omega_p \rangle \end{bmatrix} \begin{Bmatrix} \dot{x}_j \\ \dot{\eta}_p \end{Bmatrix} + \begin{bmatrix} 0 & 0 \\ 0 & \langle m_p \lambda_p \rangle \end{bmatrix}^T \begin{Bmatrix} x_j \\ \eta_p \end{Bmatrix} = \begin{Bmatrix} F_j \\ 0 \end{Bmatrix} \tag{S57}$$

Equation (S57) can be divided into two equations

$$[M_{rr}]\{\ddot{x}_j\} + [L]^T \{\ddot{\eta}_p\} = \{F_j\} \tag{S58}$$

and

$$[L]\{\ddot{x}_j\} + \langle m_p \rangle \{\ddot{\eta}_p\} + \langle 2 m_p \xi_p \omega_p \rangle \{\dot{\eta}_p\} + \langle m_p \lambda_p \rangle \{\eta_p\} = \{0\} \tag{S59}$$

Equations (S58) and (S59), when transformed in the frequency domain, give

$$[M_{rr}]\{\ddot{x}_j\} + [L]^T \{\ddot{\Pi}_p\} = \{F_j\} \tag{S60}$$

and

$$[L]\{\ddot{X}_j\} + \langle m_p \rangle \{\ddot{\Pi}_p\} + \langle 2 m_p \xi_p \omega_p \rangle \{\dot{\Pi}_p\} + \langle m_p \lambda_p \rangle \{\Pi_p\} = \{0\} \tag{S61}$$

where

$x(t) = X e^{j\omega t}$, $\ddot{X} = -\omega^2 X$,

$\eta(t) = \Pi e^{j\omega t}$, $\dot{\Pi} = j\omega \Pi$ and $\ddot{\Pi} = -\omega^2 \Pi$,

$F(t) = \hat{F} e^{j\omega t}$.

With equation (S61) we express $\{\Pi_p\}$ in $\{X_j\}$

$$m_p[-\omega^2 + 2 j \xi_p \omega_p \omega + \omega_p^2] \Pi_p = -[L_p]\{\ddot{X}_j\} \tag{S62}$$

where $[L_k] = \{\phi_{p,k}\}^T [M][\Phi_r]$ is a 1×6 vector with modal participation factors and $L_{kj} = \{\phi_{p,k}\}^T [M]\{\Phi_{r,j}\}$ is a participation factor with $k = 1,2,..., p$ and $j = 1,2,...,6$. Thus equation (S62) becomes

$$\Pi_k = -\frac{[\boldsymbol{L}_k]\{\ddot{\boldsymbol{X}}_j\}}{\boldsymbol{m}_k\omega_k^2}\left[\frac{1}{1-\left(\frac{\omega}{\omega_k}\right)^2+2\boldsymbol{j}\xi_k\frac{\omega}{\omega_k}}\right] = -\frac{[\boldsymbol{L}_k]\{\ddot{\boldsymbol{X}}_j\}}{\boldsymbol{m}_k\omega_k^2}\boldsymbol{H}_k\left(\frac{\omega}{\omega_k}\right) \qquad \text{(S63)}$$

Substituting equation (S63) into (S60) gives

$$[\boldsymbol{M}_{rr}]\{\ddot{\boldsymbol{x}}_j\}+[\boldsymbol{L}_1^T,...,\boldsymbol{L}_p^T]^T\left\{\left(\frac{\omega}{\omega_k}\right)^2\frac{[\boldsymbol{L}_k]}{\boldsymbol{m}_k}\boldsymbol{H}_k\left(\frac{\omega}{\omega_k}\right)\right\}\{\ddot{\boldsymbol{x}}_j\}=\{\hat{\boldsymbol{F}}_j\},\quad \boldsymbol{k}=1,2,...,\boldsymbol{p} \qquad \text{(S64)}$$

$$\left[[\boldsymbol{M}_{rr}]+\sum_{\boldsymbol{k}=1}^{\boldsymbol{p}}\frac{[\boldsymbol{L}_k]^T[\boldsymbol{L}_k]}{\boldsymbol{m}_k}\left\{\left(\frac{\omega}{\omega_k}\right)^2\boldsymbol{H}_k\left(\frac{\omega}{\omega_k}\right)\right\}\right]\{\ddot{\boldsymbol{x}}_j\}=\{\hat{\boldsymbol{F}}_j\} \qquad \text{(S65)}$$

We can prove that

$$[\boldsymbol{M}_{rr}]=\sum_{\boldsymbol{k}=1}^{\boldsymbol{p}}\frac{[\boldsymbol{L}_k]^T[\boldsymbol{L}_k]}{\boldsymbol{m}_k} \qquad \text{(S66)}$$

because

$$[\boldsymbol{M}_{rr}]=[\Phi_r]^T[\boldsymbol{M}][\Phi_p]([\Phi_{ip}]^T[\boldsymbol{M}_{ii}][\Phi_{ip}])^{-1}[\Phi_p]^T[\boldsymbol{M}][\Phi_r]=[\Phi_r]^T[\boldsymbol{M}][\Phi_r] \qquad \text{(S67)}$$

or

$$[\boldsymbol{M}_{rr}]=[\Phi_{ij}^T,\boldsymbol{I}]^T[\boldsymbol{M}]\begin{bmatrix}\Phi_{ip}\\0\end{bmatrix}([\Phi_{ip}]^T[\boldsymbol{M}_{ii}][\Phi_{ip}])^{-1}[\Phi_{ip}^T,0]^T[\boldsymbol{M}]\begin{bmatrix}\Phi_{ij}\\0\end{bmatrix}=[\Phi_r]^T[\boldsymbol{M}][\Phi_r]$$

(S68)

Assuming the inverse of $[\Phi_{ip}]$ exists, the modal effective mass $[\boldsymbol{M}_{eff,k}]$ is defined as follows

$$[\boldsymbol{M}_{eff,k}]=\frac{[\boldsymbol{L}_k]^T[\boldsymbol{L}_k]}{\boldsymbol{m}_k} \qquad \text{(S69)}$$

where $[\boldsymbol{L}_k]=\{\phi_{p,k}\}^T[\boldsymbol{M}][\Phi_r]$ and $\boldsymbol{m}_k=\{\phi_{p,k}\}^T[\boldsymbol{M}]\{\phi_{p,k}\}$. The summation over all modal effective masses $[\boldsymbol{M}_{eff,k}]$ will result in the rigid body mass matrix $[\boldsymbol{M}_{rr}]$ with respect to $\{\boldsymbol{x}_j\}$. Equation (S66) becomes

$$[\boldsymbol{M}_{rr}]=\sum_{\boldsymbol{k}=1}^{\boldsymbol{p}=\boldsymbol{i}}[\boldsymbol{M}_{eff,k}] \qquad \text{(S70)}$$

Therefore, equation (S65) can be rewritten as

$$\left[\sum_{\boldsymbol{k}=1}^{\boldsymbol{p}}[\boldsymbol{M}_{eff,k}]\left\{1+\left(\frac{\omega}{\omega_k}\right)^2\boldsymbol{H}_k\left(\frac{\omega}{\omega_k}\right)\right\}\right]\{\ddot{\boldsymbol{x}}_j\}=\{\hat{\boldsymbol{F}}_j\} \qquad \text{(S71)}$$

Equation (S71) can be decomposed into modal reaction forces $\{\boldsymbol{F}_{base,k}\}$

$$\sum_{k=1}^{p}\{\boldsymbol{F}_{base,k}\} = \{\hat{\boldsymbol{F}}_j\} \tag{S72}$$

where

$$\{\boldsymbol{F}_{base,k}\} = [\boldsymbol{M}_{eff,k}]\left\{1+\left(\frac{\omega}{\omega_k}\right)^2 \boldsymbol{H}_k\left(\frac{\omega}{\omega_k}\right)\right\}\{\ddot{\boldsymbol{X}}_j\} \tag{S73}$$

Equation (S73) is rather similar to equation (S37).

Interval Analysis

Basic theory of interval analysis. The design parameters in engineering are generally considered as deterministic, but there are many cases where some parameters are uncertain and therefore it is very difficult to roughly understand the product performance. These uncertain parameters come from either design tolerance, manufacture error, or even the error in assembling or working process [28-30]. When the ranges of uncertain parameters become large or the number of uncertain parameters has increased greatly, the deterministic method could not give a definite solution. To solve these problems, interval analysis [28-32] is a good method to describe all of possible results with interval expressions.

An uncertain real can be considered as a real set which contains all of the possible values within a real interval whose lower and upper bounds are all known. At the same time, a real set can be expressed as the real interval which contains the lower bound and upper bound of the real set. When the interval analysis method is utilized to solve the problems which consist of uncertain parameters, the cost will be reduced a lot. Furthermore, the interval method does not need to know the probabilistic distribution or membership function of uncertain parameters, so it can be used widely in engineering.

Let us define a real interval $[\boldsymbol{x}]$ as a connected nonempty subset of real set $\boldsymbol{R}$. It can be expressed as [29-32]

$$[\boldsymbol{x}] = [\underline{\boldsymbol{x}}, \overline{\boldsymbol{x}}] = \{\boldsymbol{x} \in \boldsymbol{R} : \underline{\boldsymbol{x}} \le \boldsymbol{x} \le \overline{\boldsymbol{x}}\} \quad \text{(S56)}$$

where $\underline{\boldsymbol{x}}$ is the lower bound of interval $[\boldsymbol{x}]$ which can also be noted as $inf([\boldsymbol{x}])$, the largest number on the left of $[\boldsymbol{x}]$; $\overline{\boldsymbol{x}}$ is the upper bound of interval $[\boldsymbol{x}]$ which can be noted as $sup([\boldsymbol{x}])$, the smallest number on right of $[\boldsymbol{x}]$. The set of all intervals over $\boldsymbol{R}$ is denoted by $\boldsymbol{IR}$ where

$$\boldsymbol{IR} = \{[\underline{\boldsymbol{x}}, \overline{\boldsymbol{x}}] : \underline{\boldsymbol{x}}, \overline{\boldsymbol{x}} \in \boldsymbol{R} : \underline{\boldsymbol{x}} \le \overline{\boldsymbol{x}}\} \quad \text{(S57)}$$

The midpoint of $[\boldsymbol{x}]$ can be expressed as

$$\boldsymbol{x}_c = \boldsymbol{mid}([\boldsymbol{x}]) = \frac{1}{2}(\underline{\boldsymbol{x}} + \overline{\boldsymbol{x}}) \quad \text{(S58)}$$

The radius of $[\boldsymbol{x}]$ is

$$\boldsymbol{rad}([\boldsymbol{x}]) = \frac{1}{2}(\overline{\boldsymbol{x}} - \underline{\boldsymbol{x}}) \quad \text{(S59)}$$

The width of $[\boldsymbol{x}]$ can be denoted by $\boldsymbol{w}([\boldsymbol{x}]) = 2\boldsymbol{rad}([\boldsymbol{x}])$. In addition, another real interval related with interval $[\boldsymbol{x}]$ can be defined as

$$[\Delta x] = [-\boldsymbol{rad}([x]), \boldsymbol{rad}([x])] \tag{S60}$$

The real interval $[x]$ can be transformed to the center form.

$$[x] = x_c + [\Delta x] \tag{S61}$$

For example

$$[x] = [2,4] = 3 + [-1,1] \quad \text{where} \quad x_c = 3,\ [\Delta x] = [-1,1] \tag{S62}$$

For further discussion, the mathematical operations are briefly introduced for the interval parameters. Interval arithmetic operations are defined on the real set *R* such that the interval expression covers all possible real results. Given the two real intervals $[x]$ and $[y]$, the operation below holds

$$[x]*[y] = [x*y : x \in [x],\ y \in [y]\} \quad \text{for} \quad * \in \{+,-,\times,\div\} \tag{S63}$$

Then, the four elementary operations are defined by

$$\begin{cases} [x]+[y] = [\underline{x}+\underline{y}, \overline{x}+\overline{y}],\ [x]-[y] = [\underline{x}-\overline{y}, \overline{x}-\underline{y}] \\ [x]\times[y] = [\min(\underline{x}\underline{y}, \underline{x}\overline{y}, \overline{x}\underline{y}, \overline{x}\overline{y}), \max(\underline{x}\underline{y}, \underline{x}\overline{y}, \overline{x}\underline{y}, \overline{x}\overline{y})] \\ \dfrac{1}{[x]} = \dfrac{1}{\overline{x}}, \dfrac{1}{\underline{x}} \quad \textbf{if } 0 \notin [x] \\ [x]\div[y] = [x]\times\dfrac{1}{[y]} \end{cases} \tag{S64}$$

Particularly

$$[x]^2 \begin{cases} [0,\ \max(\underline{x}^2, \overline{x}^2)], & \textbf{if} \quad 0 \in [x] \\ [\min(\underline{x}^2, \overline{x}^2),\ \mathbf{max}(\underline{x}^2, \overline{x}^2)] & \textbf{if} \quad 0 \notin [x] \end{cases} \tag{S65}$$

In most cases, interval arithmetic will enlarge the width of result. For example, if $[x] = [-1,1]$, then

$$4[x] - 2[x] = [-4,4] - [-2,2] = [-6,6] \tag{S66}$$

But the accurate and narrow result can be obtained by altering the operation form, namely,

$$4[x] - 2[x] = (4-2)[x] = 2\times[-1,1] = [-2,2] \tag{S67}$$

The result will be narrower when the number of the occurrences of each interval variable is as little as possible [31].

Consider a function $\boldsymbol{f}$ from $\boldsymbol{R^n}$ to $\boldsymbol{R^m}$. The interval function $[\boldsymbol{f}]$ from $\boldsymbol{IR^n}$ to $\boldsymbol{IR^m}$ is an inclusion function for $\boldsymbol{f}$ if

$$\forall [x] \in \boldsymbol{IR}^n,\ \boldsymbol{f}([x]) \subset [\boldsymbol{f}]([x]) \tag{S68}$$

where $\forall[\boldsymbol{x}]$ indicates any interval $[\boldsymbol{x}]$.

One of the most important purposes of interval analysis is to provide, for a large class of functions ***f***, inclusion functions $[\boldsymbol{f}]$ that can be evaluated reasonably and quickly such that the result interval is not too wide to engineering applications. Natural inclusion function which just needs to replace each real variable by interval variable is the simplest method to get interval result of a function. Natural inclusion function can obtain the minimal interval result if each of the variables occurs once at most in the formal function of ***f*** and the function is continuous [32].

Interval analysis of dynamic stiffness. For the sake of simplicity, the masses of *Caudipteryx*'s parts are regarded as a percentage of whole body mass and so do the stiffnesses of *Caudipteryx*'s parts attached to the main body which are considered a percentage of $\boldsymbol{K}_1$ that is supposed for the stiffness between *Caudipteryx* and Ground (Table A). The parameter $\boldsymbol{M}$ with the unit of Kg and $\boldsymbol{K}_1$ with the unit of N/mm are considered as intervals.

$$\begin{cases} [\boldsymbol{M}] = [\underline{\boldsymbol{M}}, \overline{\boldsymbol{M}}] = [3,7] \\ [\boldsymbol{K}_1] = [\underline{\boldsymbol{K}}_1, \overline{\boldsymbol{K}}_1] = [500,1500] \\ \boldsymbol{M}_c = \boldsymbol{mid}([\boldsymbol{M}]) = \frac{1}{2}(\underline{\boldsymbol{M}} + \overline{\boldsymbol{M}}) = 5\ \boldsymbol{Kg} \\ \boldsymbol{K}_{1c} = \boldsymbol{mid}([\boldsymbol{K}_1]) = \frac{1}{2}(\underline{\boldsymbol{K}}_1 + \overline{\boldsymbol{K}}_1) = 1000\ \boldsymbol{N}/\boldsymbol{mm} \\ [\Delta\boldsymbol{M}] = [-\boldsymbol{rad}([\boldsymbol{M}]), \boldsymbol{rad}([\boldsymbol{M}])] = [-2,2] \\ [\Delta\boldsymbol{K}_1] = [-\boldsymbol{rad}([\boldsymbol{K}_1]), \boldsymbol{rad}([\boldsymbol{K}_1])] = [-500,500] \\ [\boldsymbol{M}] = \boldsymbol{M}_c + [\Delta\boldsymbol{M}] = 5 + [-2,2] \\ [\boldsymbol{K}_1] = \boldsymbol{K}_{1c} + [\Delta\boldsymbol{K}_1] = 1000 + [-500,500] \end{cases} \tag{S69}$$

Table A. Percentages of masses and stiffnesses of *Caudipteryx*'s parts

Mass	Percentage		Stiffness	Percentage
$\boldsymbol{m}_1$	48%$\boldsymbol{M}$			
$\boldsymbol{m}_2$	10% $\boldsymbol{M}$		K_2	20%$\boldsymbol{K}_1$
$\boldsymbol{m}_3$	10% $\boldsymbol{M}$		K_3	20%$\boldsymbol{K}_1$
$\boldsymbol{m}_4$	4% $\boldsymbol{M}$		K_4	65%$\boldsymbol{K}_1$
$\boldsymbol{m}_5$	4% $\boldsymbol{M}$		K_5	65%$\boldsymbol{K}_1$
$\boldsymbol{m}_6$	10% $\boldsymbol{M}$		K_6	80%$\boldsymbol{K}_1$
$\boldsymbol{m}_7$	14% $\boldsymbol{M}$		K_7	80%$\boldsymbol{K}_1$
$\boldsymbol{M}$=5Kg			$\boldsymbol{K}_1$=1000N/mm	

The discrete dynamic system is governed by (Fig. 1)

$$\mathbf{M}\ddot{\boldsymbol{x}} + \mathbf{K}\boldsymbol{x} = \boldsymbol{F} \tag{S70}$$

where $\mathbf{M} = \begin{bmatrix} \boldsymbol{m}_1 & 0 & 0 & 0 & 0 & 0 & 0 \\ 0 & \boldsymbol{m}_2 & 0 & 0 & 0 & 0 & 0 \\ 0 & 0 & \boldsymbol{m}_3 & 0 & 0 & 0 & 0 \\ 0 & 0 & 0 & \boldsymbol{m}_4 & 0 & 0 & 0 \\ 0 & 0 & 0 & 0 & \boldsymbol{m}_5 & 0 & 0 \\ 0 & 0 & 0 & 0 & 0 & \boldsymbol{m}_6 & 0 \\ 0 & 0 & 0 & 0 & 0 & 0 & \boldsymbol{m}_7 \end{bmatrix}$ which is a 7×7 -mass matrix,

$$\mathbf{K} = \begin{bmatrix} \boldsymbol{k}_2 + \boldsymbol{k}_3 + \boldsymbol{k}_4 + \boldsymbol{k}_5 + \boldsymbol{k}_6 + \boldsymbol{k}_7 & -\boldsymbol{k}_2 & -\boldsymbol{k}_3 & -\boldsymbol{k}_4 & -\boldsymbol{k}_5 & -\boldsymbol{k}_6 & -\boldsymbol{k}_7 \\ -\boldsymbol{k}_2 & \boldsymbol{k}_2 & 0 & 0 & 0 & 0 & 0 \\ -\boldsymbol{k}_3 & 0 & \boldsymbol{k}_3 & 0 & 0 & 0 & 0 \\ -\boldsymbol{k}_4 & 0 & 0 & \boldsymbol{k}_1 + \boldsymbol{k}_4 & 0 & 0 & 0 \\ -\boldsymbol{k}_5 & 0 & 0 & 0 & \boldsymbol{k}_1 + \boldsymbol{k}_5 & 0 & 0 \\ -\boldsymbol{k}_6 & 0 & 0 & 0 & 0 & \boldsymbol{k}_6 & 0 \\ -\boldsymbol{k}_7 & 0 & 0 & 0 & 0 & 0 & \boldsymbol{k}_7 \end{bmatrix}$$ which is a

7×7 -stiffness matrix, $\ddot{\boldsymbol{x}} = \begin{bmatrix} \ddot{x}_1 & \ddot{x}_2 & \ddot{x}_3 & \ddot{x}_4 & \ddot{x}_5 & \ddot{x}_6 & \ddot{x}_7 \end{bmatrix}^{\boldsymbol{T}}$ which is the acceleration vector, $\boldsymbol{x} = \begin{bmatrix} \boldsymbol{x}_1 & \boldsymbol{x}_2 & \boldsymbol{x}_3 & \boldsymbol{x}_4 & \boldsymbol{x}_5 & \boldsymbol{x}_6 & \boldsymbol{x}_7 \end{bmatrix}^{\boldsymbol{T}}$ which is the displacement vector and $\boldsymbol{F}$ is the base excitation function. The answers for the homogeneous equation (S70) would be earned in terms of eigenvalues (natural frequencies) and eigenvectors (mode shapes). Therefore, matrices **M** and **K** should be rewritten as

$$\mathbf{M} = \begin{bmatrix} 0.48[\boldsymbol{M}] & 0 & 0 & 0 & 0 & 0 & 0 \\ 0 & 0.1[\boldsymbol{M}] & 0 & 0 & 0 & 0 & 0 \\ 0 & 0 & 0.1[\boldsymbol{M}] & 0 & 0 & 0 & 0 \\ 0 & 0 & 0 & 0.04[\boldsymbol{M}] & 0 & 0 & 0 \\ 0 & 0 & 0 & 0 & 0.04[\boldsymbol{M}] & 0 & 0 \\ 0 & 0 & 0 & 0 & 0 & 0.1[\boldsymbol{M}] & 0 \\ 0 & 0 & 0 & 0 & 0 & 0 & 0.14[\boldsymbol{M}] \end{bmatrix} \quad \text{(S71)}$$

and

$$\mathbf{K} = \begin{bmatrix} 0.2[\boldsymbol{k}_1] + 0.2[\boldsymbol{k}_1] + 0.65[\boldsymbol{k}_1] + 0.65[\boldsymbol{k}_1] + 0.8[\boldsymbol{k}_1] + 0.8[\boldsymbol{k}_1] & -0.2[\boldsymbol{k}_1] & -0.2[\boldsymbol{k}_1] & -0.65[\boldsymbol{k}_1] & -0.65[\boldsymbol{k}_1] & -0.8[\boldsymbol{k}_1] & -0.8[\boldsymbol{k}_1] \\ -0.2[\boldsymbol{k}_1] & 0.2[\boldsymbol{k}_1] & 0 & 0 & 0 & 0 & 0 \\ -0.2[\boldsymbol{k}_1] & 0 & 0.2[\boldsymbol{k}_1] & 0 & 0 & 0 & 0 \\ -0.65[\boldsymbol{k}_1] & 0 & 0 & [\boldsymbol{k}_1] + 0.65[\boldsymbol{k}_1] & 0 & 0 & 0 \\ -0.65[\boldsymbol{k}_1] & 0 & 0 & 0 & [\boldsymbol{k}_1] + 0.65[\boldsymbol{k}_1] & 0 & 0 \\ -0.8[\boldsymbol{k}_1] & 0 & 0 & 0 & 0 & 0.8[\boldsymbol{k}_1] & 0 \\ -0.8[\boldsymbol{k}_1] & 0 & 0 & 0 & 0 & 0 & 0.8[\boldsymbol{k}_1] \end{bmatrix}$$

(S72)

Therefore, the eigenvalues in rad/sec can be found with

$$\det\left(\mathbf{K}-\omega^2\mathbf{M}\right) = 0 \tag{S73}$$

where

$$\mathbf{M} = \begin{bmatrix} [1.44, 3.36] & 0 & 0 & 0 & 0 & 0 & 0 \\ 0 & [0.3, 0.7] & 0 & 0 & 0 & 0 & 0 \\ 0 & 0 & [0.3, 0.7] & 0 & 0 & 0 & 0 \\ 0 & 0 & 0 & [0.12, 0.28] & 0 & 0 & 0 \\ 0 & 0 & 0 & 0 & [0.12, 0.28] & 0 & 0 \\ 0 & 0 & 0 & 0 & 0 & [0.3, 0.7] & 0 \\ 0 & 0 & 0 & 0 & 0 & 0 & [0.42, 0.98] \end{bmatrix} \tag{S74}$$

and

$$\mathbf{K} = \begin{bmatrix} [1650, 4950] & -[100, 300] & -[100, 300] & -[325, 975] & -[325, 975] & -[400, 1200] & -[400, 1200] \\ -[100, 300] & [100, 300] & 0 & 0 & 0 & 0 & 0 \\ -[100, 300] & 0 & [100, 300] & 0 & 0 & 0 & 0 \\ -[325, 975] & 0 & 0 & [825, 2475] & 0 & 0 & 0 \\ -[325, 975] & 0 & 0 & 0 & [825, 2475] & 0 & 0 \\ -[400, 1200] & 0 & 0 & 0 & 0 & [400, 1200] & 0 \\ -[400, 1200] & 0 & 0 & 0 & 0 & 0 & [400, 1200] \end{bmatrix} \tag{S75}$$

The eigenvalues in rad/sec are expressed in interval vector form below

$$\begin{matrix} \bar{\boldsymbol{M}}, \underline{\boldsymbol{K}}_1 & \underline{\boldsymbol{M}}, \underline{\boldsymbol{K}}_1 & \bar{\boldsymbol{M}}, \bar{\boldsymbol{K}}_1 & \underline{\boldsymbol{M}}, \bar{\boldsymbol{K}}_1 \end{matrix}$$

$$\omega = \begin{bmatrix} 7.22418 & 11.0351 & 12.5127 & 19.1134 \\ 11.9523 & 18.2574 & 20.702 & 31.6228 \\ 13.7778 & 21.046 & 23.8639 & 36.4527 \\ 21.7618 & 33.2418 & 37.6926 & 57.5764 \\ 28.4249 & 43.4197 & 49.2334 & 75.2052 \\ 54.281 & 82.9156 & 94.0175 & 143.614 \\ 55.1034 & 84.1718 & 95.4418 & 145.79 \end{bmatrix} \tag{S76}$$

Then frequencies in Hz are shown in interval form

$$\begin{matrix} \bar{\boldsymbol{M}}, \underline{\boldsymbol{K}}_1 & \underline{\boldsymbol{M}}, \underline{\boldsymbol{K}}_1 & \bar{\boldsymbol{M}}, \bar{\boldsymbol{K}}_1 & \underline{\boldsymbol{M}}, \bar{\boldsymbol{K}}_1 \end{matrix}$$

$$\boldsymbol{f} = \begin{bmatrix} 1.14976 & 1.75629 & 1.99145 & 3.04199 \\ 1.90227 & 2.90576 & 3.29482 & 5.03292 \\ 2.19281 & 3.34957 & 3.79806 & 5.80163 \\ 3.46351 & 5.29059 & 5.99897 & 9.16357 \\ 4.52396 & 6.91047 & 7.83573 & 11.9693 \\ 8.63909 & 13.1964 & 14.9633 & 22.8569 \\ 8.76997 & 13.3964 & 15.19 & 23.2032 \end{bmatrix} \tag{S77}$$

The eigenvector matrices are

$$
\mathbf{\Phi}_{\bar{\boldsymbol{M}},\underline{\boldsymbol{K}}_1}=\begin{bmatrix}
0.33219 & 0 & 0.21615 & 0.12331 & 0.33911 & 0 & -0.1017 \\
0.52341 & 0.845154 & -0.6574 & -0.0533 & -0.0728 & 0 & 0.00502 \\
0.52341 & -0.84515 & -0.6574 & -0.0533 & -0.0728 & 0 & 0.00502 \\
0.13322 & 0 & 0.09101 & 0.05788 & 0.18406 & -1.33631 & 1.31242 \\
0.13322 & 0 & 0.09101 & 0.05788 & 0.18406 & 1.336306 & 1.31242 \\
0.36558 & 0 & 0.32367 & 0.72008 & -0.8192 & 0 & 0.02358 \\
0.3809 & 0 & 0.40408 & -0.7694 & -0.3462 & 0 & 0.0158
\end{bmatrix}
$$

$$
\mathbf{\Phi}_{\bar{\boldsymbol{M}},\bar{\boldsymbol{K}}_1}=\begin{bmatrix}
0.33219 & 0 & 0.21615 & 0.12331 & 0.33911 & 0 & -0.1017 \\
0.52341 & -0.84515 & -0.6574 & -0.0533 & -0.0728 & 0 & 0.00502 \\
0.52341 & 0.845154 & -0.6574 & -0.0533 & -0.0728 & 0 & 0.00502 \\
0.13322 & 0 & 0.09101 & 0.05788 & 0.18406 & -1.33631 & 1.31242 \\
0.13322 & 0 & 0.09101 & 0.05788 & 0.18406 & 1.336306 & 1.31242 \\
0.36558 & 0 & 0.32367 & 0.72008 & -0.8192 & 0 & 0.02358 \\
0.3809 & 0 & 0.40408 & -0.7694 & -0.3462 & 0 & 0.0158
\end{bmatrix}
$$

$$
\mathbf{\Phi}_{\underline{\boldsymbol{M}},\underline{\boldsymbol{K}}_1}=\begin{bmatrix}
0.50744 & 0 & 0.33017 & 0.18835 & 0.518 & 0 & -0.1554 \\
0.79952 & -1.291 & -1.0042 & -0.0814 & -0.1113 & 0 & 0.00767 \\
0.79952 & 1.29099 & -1.0042 & -0.0814 & -0.1113 & 0 & 0.00767 \\
0.2035 & 0 & 0.13903 & 0.08841 & 0.28116 & -2.04124 & 2.00476 \\
0.2035 & 0 & 0.13903 & 0.08841 & 0.28116 & 2.041241 & 2.00476 \\
0.55844 & 0 & 0.49442 & 1.09994 & -1.2513 & 0 & 0.03602 \\
0.58183 & 0 & 0.61724 & -1.1752 & -0.5288 & 0 & 0.02413
\end{bmatrix}
$$

$$
\mathbf{\Phi}_{\underline{\boldsymbol{M}},\bar{\boldsymbol{K}}_1}=\begin{bmatrix}
0.50744 & 0 & 0.33017 & 0.18835 & 0.518 & 0 & -0.1554 \\
0.79952 & -1.291 & -1.0042 & -0.0814 & -0.1113 & 0 & 0.00767 \\
0.79952 & 1.29099 & -1.0042 & -0.0814 & -0.1113 & 0 & 0.00767 \\
0.2035 & 0 & 0.13903 & 0.08841 & 0.28116 & 2.04124 & 2.00476 \\
0.2035 & 0 & 0.13903 & 0.08841 & 0.28116 & -2.0412 & 2.00476 \\
0.55844 & 0 & 0.49442 & 1.09994 & -1.2513 & 0 & 0.03602 \\
0.58183 & 0 & 0.61724 & -1.1752 & -0.5288 & 0 & 0.02413
\end{bmatrix}
\tag{S78}
$$

Hence, the modal participation vectors are shown in interval form

$$
\begin{array}{cccc}
\bar{\boldsymbol{M}},\underline{\boldsymbol{K}}_1 & \underline{\boldsymbol{M}},\underline{\boldsymbol{K}}_1 & \bar{\boldsymbol{M}},\bar{\boldsymbol{K}}_1 & \underline{\boldsymbol{M}},\bar{\boldsymbol{K}}_1
\end{array}
$$

$$
\boldsymbol{\Gamma} = \begin{bmatrix}
2.552737 & 1.6711588 & 2.552737 & 1.6711588 \\
0 & 0 & 0 & 0 \\
0.479452 & 0.313875 & 0.479452 & 0.313875 \\
0.122213 & 0.080007 & 0.122213 & 0.080007 \\
0.227808 & 0.149136 & 0.227808 & 0.149136 \\
0 & 0 & 0 & 0 \\
0.4322327 & 0.2829627 & 0.4322327 & 0.2829627
\end{bmatrix} \tag{S79}
$$

Both coefficient vector $\bar{\boldsymbol{L}}$ and modal participation vector $\boldsymbol{\Gamma}$ could be identical because of the generalized mass matrix. The modal effective masses $m_{eff,i}$ (where $m_{eff,i} = \frac{\bar{\boldsymbol{L}}_i^2}{\hat{\boldsymbol{m}}_{ii}}$ for mode ***i***) are shown in Table B.

Table B. Effective masses of seven-degree-of-freedom system of simplified *Caudipteryx*

Effective Mass	$\bar{\boldsymbol{M}},\underline{\boldsymbol{K}}_1$	$\underline{\boldsymbol{M}},\underline{\boldsymbol{K}}_1$	$\bar{\boldsymbol{M}},\bar{\boldsymbol{K}}_1$	$\underline{\boldsymbol{M}},\bar{\boldsymbol{K}}_1$
$\boldsymbol{m}_{eff,1}$	6.51647	2.79277	6.51647	2.79277
$\boldsymbol{m}_{eff,2}$	0	0	0	0
$\boldsymbol{m}_{eff,3}$	0.22987	0.09852	0.22987	0.09852
$\boldsymbol{m}_{eff,4}$	0.01494	0.0064	0.01494	0.0064
$\boldsymbol{m}_{eff,5}$	0.0519	0.02224	0.0519	0.02224
$\boldsymbol{m}_{eff,6}$	0	0	0	0
$\boldsymbol{m}_{eff,7}$	0.18683	0.08007	0.18683	0.08007
Total in Kg	7	3	7	3

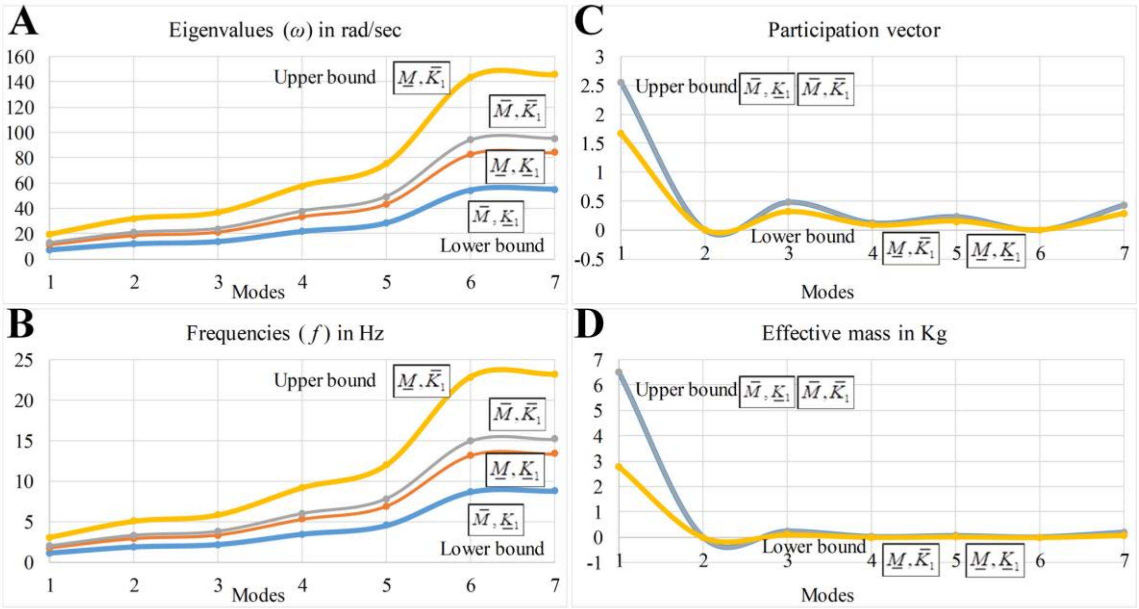

Figure G. Interval analysis of modal effective mass parameters. **A**, the eigenvalues in rad/sec are expressed in interval vector form. **B**, the frequencies in Hertzs are deduced in interval form. **C**, participation vectors are drawn in interval form and **D**, effective mass in interval form are given. In all cases, lower bound is in $\bar{\boldsymbol{M}}, \underline{\boldsymbol{K}}_1$ and upper one is in $\underline{\boldsymbol{M}}, \bar{\boldsymbol{K}}_1$.

***Caudipteryx* velocity**

When *Caudipteryx* ran at a constant velocity $\boldsymbol{V}$ and frequency $\boldsymbol{f}$ for a certain distance $\boldsymbol{L}$ with footsteps $\boldsymbol{N_f}$ in a time period $\boldsymbol{T}$, the running frequency can be calculated as $\boldsymbol{f} = \boldsymbol{N}_f / \boldsymbol{T}$. The running velocity is $\boldsymbol{V} = \boldsymbol{L}/\boldsymbol{T}$ and the step length is $\boldsymbol{L}_s = \boldsymbol{L}/(2\times \boldsymbol{N}_f)$,

Therefore the velocity can be deduced $\boldsymbol{V} = 2\times \boldsymbol{L}_s \times \boldsymbol{f}$.

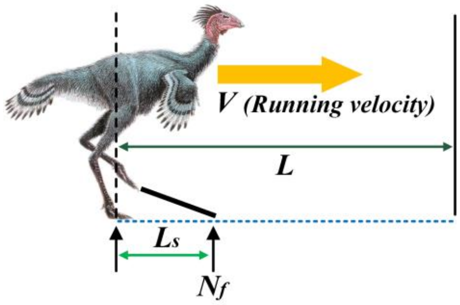

Figure H. Schematic motion of *Caudipteryx*.

By measuring the fossil, we estimate that the step length of *Caudipteryx* is about 50 cm. It means that if the running frequency of *Caudipteryx* was 2 Hz, the velocity might be 2 m/s. Therefore, we can calculate the speed of *Caudipteryx* in any modes and especially in effective modes.

According to interval analysis method, if we define lower and upper limit for the step length with the unit of centimeter:

$$\boldsymbol{L}_s = [\underline{\boldsymbol{L}}_s, \overline{\boldsymbol{L}}_s] = [30, 70] \qquad \text{(S80)}$$

Then the interval velocities of *Caudipteryx* at the first fourth modes according to the Table E in S1 Table are given in Table C.

Table C. Interval velocities of *Caudipteryx* at the first fourth modes

Mode Number	Frequency (Hertz)	Effective Mass (gram) in Y-Axis	Interval Velocity $\boldsymbol{V} = [\underline{\boldsymbol{V}}, \overline{\boldsymbol{V}}]$ (m/s)
1	1.9894	1.29E-06	[1.2, 2.78]
**2**	**2.5851**	**1623.33**	[1.5, 3.62]
3	5.7887	1554.2	[3.4, 8.1]
4	5.8629	377.657	[3.51, 8.2]

Let us take the second mode in Table C as an example. The interval velocity [1.5, 3.62] means that the flapping motion of the wings of a *Caudipteryx* was easily exited when it ran at the speed from 1.5m/s to 3.62m/s even if the variations of step length were considered.

References

1. Thomson, W.T. & Dahleh, M.D. *THEORY OF VIBRATION WITH APPLICATIONS*. DOI 10.1007/978–1–4899–6872–2 (1993).
2. Skelton, R.E. & Hughes, P.C. Modal cost analysis for linear matrix-second-order systems, *Journal of Dynamic Systems, Measurement, and Control*, **102,** 151-158 (1980).
3. Moore, B.C. Principal component analysis in linear systems: controllability, observability, and model reduction. *IEEE Transactions on Automatic Control* **AC-26**, 17-32 (1981).
4. Doran, A.L. & Mingori, D.L. Model reduction by cost decomposition: implications of coordinate selection, *The Journal of the Astronautical Sciences* **31(3)**, 415-428 (1983).
5. Pernebo, L. & Silverman, L.M. Model reduction via balanced state space representations, *IEEE Transactions on Automatic Control* **AC-28**, 240-242 (1983).
6. Gregory, C.Z. Reduction of large flexible spacecraft models using internal balancing theory, *Journal of Guidance, Control, and Dynamics* **7**, 725-732 (1984).
7. Kim, Y. & Junkins, J.L. Measure of controllability for actuator placement, *Journal of Guidance, Control, and Dynamics* **14(5),** 895-902 (1991).
8. Blelloch, P.A. & Carney, K.S. Modal selection in structural dynamics, *7th International Modal Analysis Conference, Las Vegas, Nevada* (1989).
9. Kammer, D.C. & Triller, M.J. Ranking the dynamic importance of fixed interface modes using a generalization of effective mass. *International Journal of Analytical and Experimental Modal Analysis* **9(2)**, 77-98 (1994).
10. Plesseria, J.Y., Rochus, P. & Defise, J.M. Effective modal masses, 5éme Congrès National de Mécanique Théorique et Appliquée, Louvain-la-Neuve, 23 & 24 mai (2000).
11. Girard, A. Réponse des structures à un environnement basse fréquence. Note technique du CNES
12. Imbert, J.F. & Mamode, A. La masse effective, unconcept important pour la caractérisation dynamique es structures avec excitation de la base. *Mécanique, matériaux, électricité*, 342-354 (1978).
13. Defise, J.M. Dynamique d'une expérience spatiale: analyses et vérification. 3ème Congrès National Belge de Mécanique Théorique et Appliquée, 253-256, Université de Liège (1994).
14. Rochus, P., Defise, J.M., Plesseria, J.Y., Hault, F. & Janssen, G. Effective modal parameters to evaluate structural stresses. *Proceedings European Conference on Spacecraft Structures, Materials and Mechanical Testing*, Braunschweig, **ESA SP-428,** 437-442 (1999).
15. Girard, A. & Roy, N.A. Modal effective parameters in structural dynamics. *Revue Européenne des éléments finis* **6**, 233-254 (1997).
16. Papadrakakis, M., Lagaros, N. & Plevris, V. Optimum design of structures under seismic loading, *European Congress on Computational Methods in Applied Sciences and Engineering, Barcelona* (2000).
17. Irvine, T. The generalized coordinate method for discrete systems. *Vibrationdata* (2000).
18. Irvine, T. Bending frequencies of beams, rods, and pipes, Rev M, *Vibrationdata* (2010).

19. Irvine, T. Rod response to longitudinal base excitation, steady-state and transient, Rev B, *Vibrationdata* (2009).
20. Irvine, T. Longitudinal vibration of a rod via the finite element method, Revision B, *Vibrationdata* (2008).
21. Thomson, W.T. Theory of vibration with applications. 2nd Edition, Prentice Hall, New Jersey (1981).
22. Wijker, J.J. Spacecraft structures (2008).
23. Appel, S. Calculation of modal participation factors and effective mass with the large mass approach. *Fokker Space Report* FSS-R-92-0027 (1992).
24. Craig Jr, R.R. & Bampton M.C.C. Coupling of substructures for dynamic analysis, *AIAA Journal* **6 (7)**, 1313–1319 (1968).
25. Shunmugavel, P. Modal effective masses for space vehicles. *Rockwell Space Systems Division, Downey, California AIAA* 95-125 (1995).
26. Witting M. & Klein M. Modal selection by means of effective masses and effective modal forces an application example, *Proc. Conference on Spacecraft Structures, Materials & Mechanical Testing*, 2729 March, ESA SP-386 (1996).
27. Craig Jr, R.R. Structural dynamics. John Wiley & Sons (1981).
28. Wang, J.Y., Zhao, J.S., Chu, F.L. & Feng, Z.J. Dynamic stiffness of a lift mechanism for forklift truck, *Proc IMechE Part C: J Mechanical Engineering Science* DOI: 10.1177/0954406212446901, **0(0)** 1–16 (2013).
29. Moore, R.E. Interval analysis. New York: Prentice-Hall (1996).
30. Wu, J.L., Zhang Y.Q., Chen, L.P. & *et al*. Uncertain analysis of vehicle handling using interval method, *Int J Vehicle Des* **56(1–4)** 81–105 (2011).
31. Jaulin, L. Applied interval analysis: with examples in parameter and state estimation, robust control and robotics. New York: Springer (2001).
32. Moore, R.E. Methods and applications of interval analysis. Philadelphia: SIAM (1979).
