# Supplementary material for: Identification of avian flapping motion from non-volant winged dinosaurs based on modal effective mass analysis
Source: PLoS Comput Biol. 2019 May 2;15(5):e1006846. doi: 10.1371/journal.pcbi.1006846 (PMC6497222; doi:10.1371/journal.pcbi.1006846)
Supplement: S1 Table — (PDF) [file pcbi.1006846.s012.pdf]

Tables

Identification of avian flapping motion from non-volant winged dinosaurs based on modal effective mass analysis

Yaser Saffar Talori[1], Jing-Shan Zhao[1*], Yun-Fei Liu[1], Wen-Xiu Lu[1], Zhi-Heng Li[2] and Jingmai Kathleen O'Connor[2]

*Jing-Shan Zhao
Email: jingshanzhao@mail.tsinghua.edu.cn

This file includes:

Tables A to F

Table A. Parameters of *Caudipteryx zoui* (BPM 0001).

Measurements of the *Caudipteryx* model		
	**Length (mm)**	**Mass (gram)**
**Maximum skull length**	91	500
**Maximum skull width**	59	
**Total length of cervical vertebrae**	176	
**Body length**	215	2400
**Maximum body height**	165	
**Humerus length**	72	1000
**Ulna length**	62	
**Radius length**	59	
**Manus**	91	
**Femur length**	145	400
**Tibia**	188	
**Fibula**	188	
**Length of tail**	221	700

Table B. Mass and stiffness distribution of *Caudipteryx*.

**Mass**	**Value (Kg)**	**Stiffness**	**(N/mm)**
m_1	2.400	k_1	1000
m_2	0.500	k_2	200
m_3	0.500	k_3	200
m_4	0.200	k_4	650
m_5	0.200	k_5	650
m_6	0.500	k_6	800
m_7	0.700	k_7	800
**Total mass**	**5**		

Table C. Natural frequencies and effective masses of simplified Seven-degree-of-freedom system of *Caudipteryx*.

**Natural Frequencies**	**(Hz)**	**Effective Mass**	**(Kg)**
f_1	**1.9239**	$m_{eff,1}$	*4.6546*
f_2	3.1831	$m_{eff,2}$	0
f_3	3.6693	$m_{eff,3}$	0.1642
f_4	5.7956	$m_{eff,4}$	0.0107
f_5	7.57	$m_{eff,5}$	0.0371
f_6	14.456	$m_{eff,6}$	0
f_7	14.675	$m_{eff,7}$	0.1334
		**Total**	**5 Kg**

Table D. Eight excessive assumed mass distribution in the format of models A, B, ..., I, and model D is the actual one.

Assumed Mass Body (Gram)									
**Mass**	A	B	C	**D**	E	F	G	H	I
m_1	960	1440	1920	**2400**	2880	3360	3840	4320	4800
m_2	200	300	400	**500**	600	700	800	900	1000
m_3	200	300	400	**500**	600	700	800	900	1000
m_4	80	120	160	**200**	240	280	320	360	400
m_5	80	120	160	**200**	240	280	320	360	400
m_6	200	300	400	**500**	600	700	800	900	1000
m_7	280	420	560	**700**	840	980	1120	1260	1400
**Total**	**2000**	**3000**	**4000**	**5000**	**6000**	**7000**	**8000**	**9000**	**10000**

Table E. Computer simulation for the first twenty natural frequencies and modal effective mass of *Caudipteryx*.

Eigenvalues Output				Effective Mass Output (gram)		
Mode Number	Eigen Value	FREQUENCY (Rad/Time)	FREQUENCY (Cycles/Time)	X-Axis	Y-Axis	Z-Axis
**1**	156.25	12.5	**1.9894**	7.28E-32	1.29E-06	4.83E-30
**2**	263.83	16.243	**2.5851**	3.25E-31	**1623.33**	8.46E-33
**3**	1322.9	36.371	**5.7887**	2.80E-30	**1554.2**	1.30E-30
**4**	1357	36.838	**5.8629**	7.77E-31	**377.657**	1.29E-31
5	3270.3	57.187	9.1015	2.93E-30	1.99E-07	1.14E-28
6	4397.7	66.315	10.554	4.77E-31	0.05959	2.93E-29
7	5631	75.04	11.943	1.66E-30	4.10E-07	9.46E-29
8	7848.7	88.593	14.1	1.08E-28	4.81E-09	1.02E-33
**9**	11892	109.05	**17.356**	4.44E-28	**117.041**	9.09E-28
10	23410	153	24.351	4.22E-30	13.2134	1.05E-28
11	24754	157.33	25.041	1.15E-28	43.6661	6.34E-28
12	33380	182.7	29.078	2.48E-28	2.27E-07	4.62E-27
**13**	42378	205.86	**32.764**	4.91E-27	**351.593**	3.44E-27
14	55747	236.11	37.578	1.34E-25	2.05E-07	1.05E-24
15	71139	266.72	42.45	6.55E-21	5.11E-07	4.55E-22
**16**	95222	308.58	**49.112**	7.25E-19	**108.981**	4.37E-19
17	106000	326.05	51.892	1.17E-17	23.5505	1.35E-17
**18**	111000	333.47	**53.074**	1.40E-17	**265.408**	1.50E-17
19	126000	355.47	56.574	2.71E-17	8.00E-11	8.70E-19
20	143000	377.59	60.096	2.32E-17	4.18081	1.31E-17
**Total**				7.67E-17	**4482.88**	4.30E-17

Table F. Computer simulation of the first twenty natural frequencies and modal effective mass in Y-axis for the models A, B, …, and I.

	A		B		C		D		E		F		G		H		I	
**Modes**	***F***	***EM***	***F***	***EM***	***F***	***EM***	***F***	***EM***	***F***	***EM***	***F***	***EM***	***F***	***EM***	***F***	***EM***	***F***	***EM***
**1**	3.15	0.00	2.57	0.00	2.22	0.00	**1.99**	0.00	1.82	0.00	1.68	0.00	1.57	0.00	1.48	0.00	1.41	0.00
**2**	**4.09**	**649.38**	**3.34**	**974.03**	**2.89**	**1298.68**	**2.59**	**1623.33**	**2.36**	**1947.99**	**2.18**	**2272.64**	**2.04**	**2597.29**	**1.93**	**2921.94**	**1.83**	**3246.59**
**3**	**9.15**	**621.27**	**7.47**	**932.24**	**6.47**	**1243.22**	**5.79**	**1554.20**	**5.28**	**1865.18**	**4.89**	**2176.16**	**4.58**	**2487.14**	**4.31**	**2798.12**	**4.09**	**3109.10**
**4**	9.27	151.47	7.57	226.87	6.56	302.26	**5.86**	**377.66**	5.35	453.05	4.96	528.45	4.63	603.84	4.37	679.24	4.15	754.63
**5**	14.39	0.00	11.75	0.00	10.18	0.00	9.10	0.00	8.31	0.00	7.69	0.00	7.20	0.00	6.78	0.00	6.44	0.00
**6**	16.69	0.02	13.63	0.04	11.80	0.05	10.55	0.06	9.63	0.07	8.92	0.08	8.34	0.10	7.87	0.11	7.46	0.12
**7**	18.88	0.00	15.42	0.00	13.35	0.00	11.94	0.00	10.90	0.00	10.09	0.00	9.44	0.00	8.90	0.00	8.45	0.00
**8**	22.29	0.00	18.20	0.00	15.76	0.00	14.10	0.00	12.87	0.00	11.92	0.00	11.15	0.00	10.51	0.00	9.97	0.00
**9**	27.44	46.84	22.41	70.24	19.40	93.64	**17.36**	**117.04**	15.84	140.44	14.67	163.84	13.72	187.25	12.94	210.65	12.27	234.05
**10**	38.50	5.28	31.44	7.92	27.23	10.57	24.35	13.21	22.23	15.86	20.58	18.50	19.25	21.15	18.15	23.80	17.22	26.44
**11**	39.60	17.46	32.33	26.20	28.00	34.93	25.04	43.67	22.86	52.40	21.16	61.14	19.80	69.87	18.66	78.60	17.71	87.34
**12**	45.97	0.00	37.54	0.00	32.51	0.00	29.08	0.00	26.55	0.00	24.58	0.00	22.99	0.00	21.67	0.00	20.56	0.00
**13**	51.80	140.64	42.30	210.96	36.63	281.28	**32.76**	**351.59**	29.91	421.91	27.69	492.23	25.90	562.55	24.42	632.86	23.17	703.18
**14**	59.42	0.00	48.52	0.00	42.01	0.00	37.58	0.00	34.30	0.00	31.76	0.00	29.71	0.00	28.01	0.00	26.57	0.00
**15**	67.12	0.00	54.80	0.00	47.46	0.00	42.45	0.00	38.75	0.00	35.88	0.00	33.56	0.00	31.64	0.00	30.02	0.00
**16**	77.65	43.64	63.40	65.42	54.91	87.20	**49.11**	**108.98**	44.83	130.76	41.51	152.54	38.83	174.32	36.61	196.10	34.73	217.88
**17**	82.06	9.30	67.00	14.05	58.02	18.80	51.89	23.55	47.37	28.30	43.86	33.05	41.02	37.80	38.68	42.55	36.69	47.29
**18**	83.92	106.23	68.52	159.29	59.34	212.35	**53.07**	**265.41**	48.45	318.47	44.86	371.53	41.96	424.59	39.56	477.64	37.53	530.70
**19**	89.45	0.00	73.04	0.00	63.25	0.00	56.57	0.00	51.65	0.00	47.81	0.00	44.73	0.00	42.17	0.00	40.00	0.00
**20**	95.02	1.67	77.58	2.51	67.19	3.34	60.10	4.18	54.86	5.02	50.79	5.86	47.51	6.69	44.79	7.53	42.49	8.37

Models A, B, C, ..., I   Modes 1, 2, 3, ..., 20   *f*=Frequency in Hertzs   EM=Effective Mass (Y-Axis) in Grams
